# The Reduction of Carbonyl Compounds with Dicyclopentylzinc: A New Example of Asymmetric Amplifying Autocatalysis

**DOI:** 10.3390/ijms242317048

**Published:** 2023-12-01

**Authors:** Elena Sh. Saigitbatalova, Liliya Z. Latypova, Almaz A. Zagidullin, Almira R. Kurbangalieva, Ilya D. Gridnev

**Affiliations:** 1 Biofunctional Chemistry Laboratory, A. Butlerov Institute of Chemistry, Kazan Federal University, 18 Kremlyovskaya Street, 420008 Kazan, Russia; 2 A. E. Arbuzov Institute of Organic and Physical Chemistry, FRC Kazan Scientific Center of RAS, 8 Arbuzov Street, 420088 Kazan, Russia; 3 N. D. Zelinsky Institute of Organic Chemistry, Leninsky Prosp. 47, 119991 Moscow, Russia

**Keywords:** Soai reaction, chirality amplification, dicyclopentylzinc, DFT, heterocyclic ketones, asymmetric autocatalysis

## Abstract

A previously unknown reduction of carbonyl compounds with dicyclopentylzinc is reported. Aldehydes react in mild conditions yielding corresponding primary alcohols and cyclopentene. Although cyclohexanone and acetophenone are inert to dicyclopentylzinc, a variety of heterocyclic ketones reacted readily, yielding reasonable to high yields of corresponding secondary alcohols. When the reaction was catalyzed with (–)-(1*R*,2*S*)-ephedrine, 3-acetylpyridine (**10**) resulted in a high yield of (*S*)-1-(pyridin-3-yl)ethanol (**19**) with >99% ee. 5-Acetyl-2-bromopyridine (**11**) also provided the corresponding optically active alcohol **20**, albeit with a much lower optical yield. When 10% of **19** with 92% ee was used as an autocatalyst, 55% yield of the same compound was obtained, with 95% ee and 96% ee in two independent experiments. A three-stage reaction sequence starting from “no chirality” reaction yielded **19** with 6% ee. Thus, amplifying autocatalysis was detected in the reaction of ketone **10** with dicylopentylzinc.

## 1. Introduction

The phenomenon of autocatalytic autoamplification so far has only been observed in the Soai reaction [1,2], i.e., the reaction of diisopropylzinc with pyrimidine aldehydes with a strictly defined structure. Despite the vigorous interest among researchers to investigate this unusual transformation, which, among other specific features, leads to the spontaneous generation of chirality [3,4,5,6], the extension of the reaction scope has had only very limited success. A pyridinic aldehyde with the same linker and anchor was also found to undergo an autocatalytic reaction with diisopropylzinc accompanied by the amplification of chirality [7,8]. Hence, new examples of such transformations are needed for a better understanding of the intrinsic mechanisms of asymmetric autoamplification and the spontaneous generation of chirality.

So far, only diisopropylzinc has been successfully applied as an alkylating reagent in the Soai reaction. Structurally, a cyclopentyl group is close to the isopropyl group, so we decided to study dicyclopentylzinc in the Soai-type reaction.

To the best of our knowledge, dicyclopentylzinc has been previously applied in the nucleophilic alkylation of different benzyl and heteroaryl halides [9,10,11], the regioselective C–H bond functionalization of acridine [12], 1,6-conjugate addition to paraquinone methides in a microreactor [13], and the nickel-catalyzed alkylation of electron-poor cyclobutanone oximes [14]. With methyllithium, dicyclopentylzinc forms mixed trialkylzincate nucleophiles, which can be further used in conjugate addition to cycloalkenones [15].

Moreover, the enantioselective alkylation of cyclopropanecarboxaldehyde, furfural, and benzaldehyde with dicyclopentylzinc in the presence of a chiral catalyst was reported [16,17]. We are not aware of any reactions of dicyclopentylzinc with ketones described in the literature.

Here, we report the reduction of carbonyl compounds with dicyclopentylzinc, the enantioselective reduction of 3-acetylpyridines catalyzed with (–)-(1*R*,2*S*)-ephedrine, and asymmetric autocatalytic amplification in the reaction of dicyclopentylzinc with 3-acetylpyridine.

## 2. Results

### 2.1. Discovery of the Reducing Properties of Dicyclopentylzinc

**Scheme 1:**
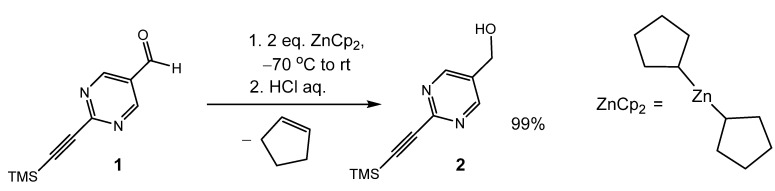
Reduction of the pyrimidine aldehyde **1** with dicyclopentylzinc.

We reckoned that since a cyclopentyl group is the closest analog of an isopropyl group, it would be worth investigating dicyclopentylzinc in the reaction with one of the most effective substrates of the Soai reaction, viz., the pyrimidinic aldehyde **1**. Unexpectedly, it was found that instead of the desired alkylation product, the primary alcohol **2** formed quantitatively. Cyclopentene was identified as another reaction product (Scheme 1). This result prompted us to investigate further reducing properties of dicyclopentylzinc.

### 2.2. Reactions of Dicyclopentylzinc with Aldehydes

Reactions of aldehydes with dicyclopentylzinc were carried out at an equimolar ratio in hexane at room temperature. No alkylation products were observed, and corresponding alcohols as the exclusive products were obtained. The results of the reduction of representative aldehydes with dicyclopentylzinc are summarized in Table 1. Entries 1–4 show that the reaction is effective for various aromatic aldehydes. Both cyclopentyl groups can be used for the reduction (Entry 2), whereas in comparing the reaction conditions in Entries 4 and 5, one can conclude that an electron-acceptor substituent has an accelerating effect (Entry 5), while an electron-donating methoxy group decreases the reactivity (Entry 4).

Aliphatic aldehydes demonstrate lower reactivity (Entries 5 and 6), but 2-, 3-, and 4-pyridinecarboxaldehydes result in excellent yields of the reduction products (Entries 7–9). It is worth noting that Ishihara reported the catalytic alkylation of furfural and cyclopropanecarboxaldehyde with generated in situ dicyclopentylzinc [16].

Control experiments were performed with diisopropylzinc. With hexanal, no appreciable amount of the reduction product was obtained. In fact, the alkylation process was very slow, yielding a multispot reaction mixture. Benzaldehyde in the reaction with 1 eq. of ZnPr*^i^*_2_ after 26 h at rt in hexane yielded 26% of the alkylation product, and 74% of the starting material was recovered.

### 2.3. Reactions of Dicyclopentylzinc with Ketones

Acetophenone and cyclohexanone do not react with dicyclopentylzinc. Numerous products of aldol condensation were detected in the first case. Only starting materials were observed in the reaction of 3-acetylindole with dicyclopentylzinc at room temperature as well as after heating for 7 h at 60 °C.

**Scheme 2:**
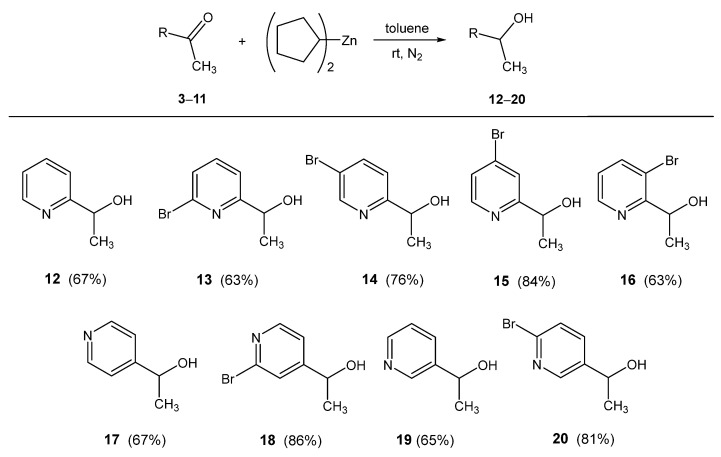
Reactions of pyridinic ketones **3**–**11** with dicyclopentylzinc.

**Scheme 3:**
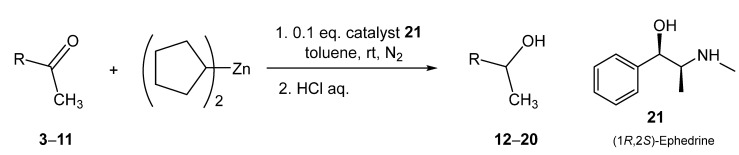
Reactions of acetylpyridines **3**–**11** with dicyclopentylzinc in the presence of catalyst.

A series of differently substituted pyridine ketones **3**–**11** were involved in the reactions with dicyclopentylzinc (Scheme 2 and Scheme 3, Table 2). Ketones **3**–**11** with pyridinic core were readily reduced to the corresponding secondary alcohols **12**–**20**. According to the NMR spectra of crude reaction mixtures, 4–15% of starting ketones remained unreacted, although the organozinc reagent was used in excess (3–5 equivalents). No signals of any alkylation products were observed in NMR spectra.

Since an asymmetric carbon atom is generated in this transformation, we decided to check the possibility of the catalytic asymmetric reduction using (1*R*,2*S*)-ephedrine (**21**) as a catalyst. The analysis of the obtained data led us to conclude that the asymmetric reaction was possible, but solely for 3-acetylpyridines **10** and **11**, which showed enantiomeric excess (Table 2) since the secondary alcohols obtained from 2-acetylpyridines **3**–**7** and 4-acetylpyridines **8** and **9** were not optically active. It was previously reported that the enantioselective reduction of isomeric acetylpyridines **3**, **8,** and **10** can be achieved using chiral lithium borohydride modified with *N*,*N*′-dibenzoylcystine and ethanol [18].

The best ee was obtained in the case of 3-acetylpyridine (**10**) (Entries 2 and 3, Table 2), which prompted us to study the asymmetric reduction of ketone **10** in more detail. Two independent experiments under similar reaction conditions reproduced the high ee value.

### 2.4. Computational Studies of the Reduction of Pyridinic Ketones with Dicyclopentylzinc

**Scheme 4:**
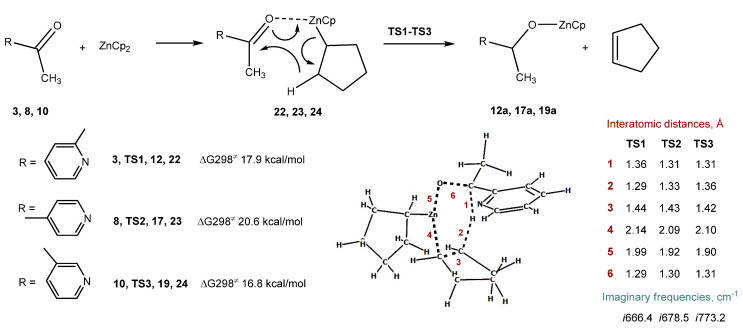
Computational data for the transition states **T1**–**T3**.

In order to study distinctive features of the reaction of dicyclopentylzinc with ketones, we located the transition states for the reduction of the ketones **3**, **8**, and **10** (Scheme 4).

Computations attest to a synchronous transformation through a six-membered transition state. The lowest activation barrier was computed for the reduction of 3-acetylpyridine **10**. Although we are unaware of the synthetically meaningful reactions of this type, similar transformations are frequently observed as side processes in the reactions of hindered Grignard reagents with carbonyl compounds [19,20].

### 2.5. Asymmetric Amplifying Autocatalysis in the Reaction of Dicyclopentylzinc with 3-Acetylpyridine

Following the protocol of the famous Soai reaction [1], we used optically active (*S*)-1-(pyridin-3-yl)ethanol (**19**) as a catalyst for the reduction of 3-acetylpyridine (**10**) with dicyclopentylzinc (Table 2). To our delight, a clean formation of **19** with the increased ee was observed. For example, when 16 mg of (*S*)-1-(pyridin-3-yl)ethanol **19** with 92% ee was used as a catalyst for the reaction of 156 mg of 3-acetylpyridine **10** with dicyclopentylzinc, 87 mg of (*S*)-1-(pyridin-3-yl)ethanol **19** with 96% ee was obtained.

Interestingly, the same alcohol ((*S*)-(–)-(**19**)) with 42% ee was used as an asymmetric autocatalyst in the methylation reaction between pyridine-3-carbaldehyde and dimethylzinc, resulting in the formation of product **19** with 7% ee of the same absolute configuration [21].

**Scheme 5:**
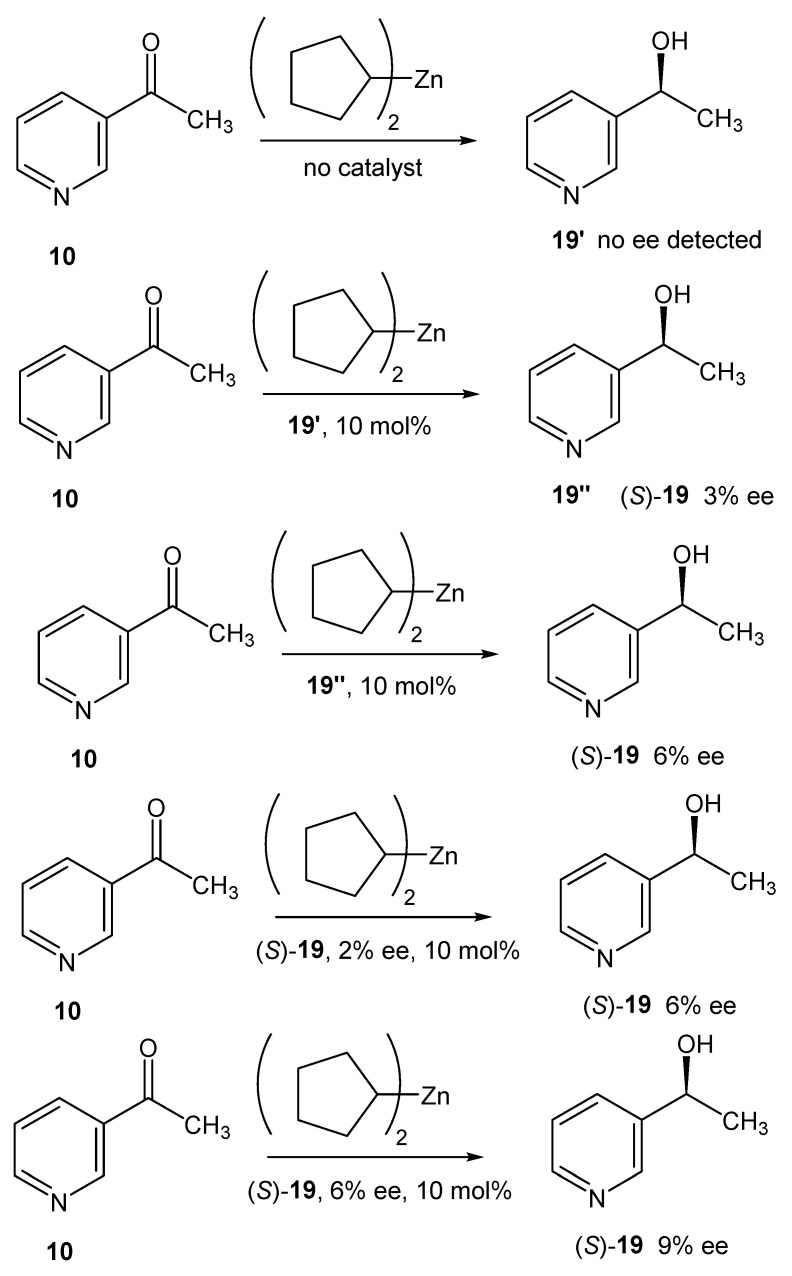
Asymmetric amplifying autocatalysis in the reaction of ketone **10** with dicyclopentylzinc.

Searching for further support for our observation, we applied the three-stage reaction sequence developed by Soai et al. [1] (Scheme 5). Product **19**′ obtained in a non-catalytic reaction was used as a catalyst in the second run, yielding **19**″ of 3% ee. This was in turn used as a catalyst in the third reaction to provide (*S*)-**19** with 6% ee. The results of two further experiments with 2% ee and 6% ee catalysts are in line with the results of the three-stage reaction sequence.

Thus, we conclude that asymmetric amplifying autocatalysis plays a role in the reaction of 3-acetylpyridine (**10**) with dicyclopentylzinc, although the level of this effect is significantly lower than that in the reactions of **1** and structurally similar aldehydes.

### 2.6. Computational Search of the Possible Mechanism of Autoamplification

Elucidating the mechanism of asymmetric amplification is a difficult task, requiring extensive studies of the equilibria in the reaction pool and computations based on experimental evidence [22]. We will pursue this task in our further studies.

**Scheme 6:**
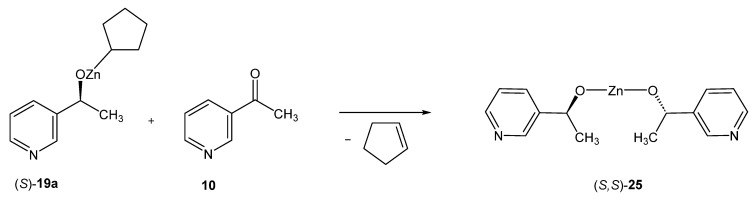
The computed enantioselective reaction of alcoholate **19a** with 3-acetylpyridine (**10**).

**Figure 1:**
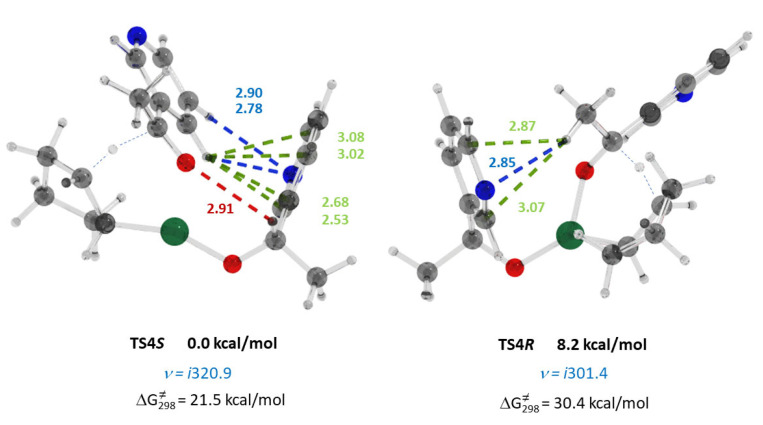
Computed structures of transition states for enantioselective reduction of 3-acetylpyridine **10** with (*S*)-alcoholate **19a**. The greater stability of **TS4*S*** is due to a much more extensive framework of the stabilizing non-covalent interactions: lime, C–H–π; blue, C–H–N; red, C–H–O. Red ball—oxygen atom, green ball—zinc atom, blue ball—nitrogen atom.

Nevertheless, preliminary computations revealed a highly enantioselective reaction between a monomeric alcoholate (*S*)-**19a** and 3-acetylpyridine (**10**), yielding bis-alcoholate (*S*,*S*)-**25** and cyclopentene (Scheme 6 and Figure 1).

Computations allowed us to predict very high enantioselectivity in favor of the formation of (*S*,*S*)-**25** through the transition state **TS4*S*** since the transition state **TS4*R*** was computed to be 8.2 kcal/mol less stable.

On the other hand, the activation barrier was computed to be around 5 kcal/mol, higher than that for the reaction of ketone **10** with dicyclopentylzinc (Scheme 4).

These results correspond roughly to the experimental data: The high level of stereodiscrimination in this system is leveled by a much faster background reaction with more active dicyclopentylzinc. Further mechanistic studies may be able to help overcome this issue.

Thus, we found a new example of asymmetric amplifying autocatalytic reaction. So far, we have obtained only (***S***)-**19** in our experiments. Hence, currently, we cannot claim a spontaneous chirality generation until the random generation of handedness is proved as in the case of the Soai reaction [4,5,6]. Further synthetic and mechanistic studies of this remarkable transformation are underway in our laboratories.

## 3. Discussion

To the best of our knowledge, the reaction of dicyclopentylzinc with 3-acetylpyridine catalyzed by its own optically active product is only the second clear example of asymmetric autoamplification ever presented since the Soai reaction was discovered 30 years ago and actively investigated thereafter [1,2]. Similarly to the pioneering discovery of Soai, the newly found asymmetric amplifying autocatalytic reaction involves the participation of an organozinc reagent that is structurally similar to diisopropylzinc operating in the Soai reaction. This is an important analogy that will certainly help in further experimental and computational mechanistic investigations aimed at providing a deeper understanding of this rare phenomenon. On the other hand, these two reactions are intrinsically different in terms of the nature of chemical transformation—alkylation in the Soai reaction vs. reduction in the reaction reported here. This fact improves the prognoses for possible findings of other examples of asymmetric autoamplification. In addition, unlike the sophisticated substrates of the Soai reaction, 3-acetylpyridine is a commercially available reagent that opens the door for extensive studies and possible practical applications of asymmetric autoamplification. We are actively working according to these considerations in our laboratories.

## 4. Materials and Methods

### 4.1. Experimental Details

Dicyclopentylzinc was prepared according to the known procedure [23]. All solvents were purified and distilled using standard procedures. Analytical thin-layer chromatography (TLC) was carried out on Sorbfil PTLC-AF-A-UF plates using UV light (254 nm) as the visualizing agent. Silica gel 60A (Acros Organics, Geel, Belgium, 400–230 mesh, 0.040–0.063 mm) was used for open column chromatography. Melting points were recorded with a Boëtius melting point instrument and are uncorrected. NMR spectra were measured on a Bruker Avance III 400 spectrometer at 400.17 MHz (^1^H) and 100.62 MHz (^13^C), and a Bruker Avance III 500 spectrometer at 500.1 MHz (^1^H) and 125.8 MHz (^13^C) at 20 °C in deuterated chloroform and benzene. The chemical shifts (δ) are expressed in parts per million (ppm) and are calibrated using residual undeuterated solvent peak as an internal reference (CDCl_3_: δ_H_ 7.26, δ_C_ 77.16; C_6_D_6_: *δ*_H_ 7.16, *δ*_C_ 128.06). All coupling constants (*J*) are reported in Hertz (Hz), and multiplicities are indicated as follows: s (singlet), d (doublet), dd (doublet of doublets), qd (quartet of doublets), and m (multiplet). The enantiomeric excess (ee) measurements were performed via HPLC analysis on an HPLC system equipped with chiral stationary-phase column Chiralpak (OD-H), detection at 220 nm. Synthetic procedures and characterization details for all compounds can be found in the Supplementary Materials (Figures S1–S13). Spectral data of synthesized compounds are consistent with the literature [24,25,26,27,28,29,30,31,32,33,34,35,36,37,38,39].

### 4.2. Computational Details

Geometry optimizations were performed without any symmetry constraints (C1 symmetry) using the ωB97XD functional [40] as implemented in the Gaussian16 software revision C.01 package [41]. Frequency calculations were carried out to confirm the nature of the stationary points, yielding one imaginary frequency for all transition states (TSs) and zero for all minima. Constrained energy hypersurface scans were conducted to investigate the molecular reactivity and to locate viable reaction channels. Where low-lying barriers were estimated, frequency calculations were performed at the crude saddle points, and the obtained force constants were used to optimize the transition state structures employing the Berny algorithm [42]. All atoms were described with a 6–31 G(d,p) basis set in the geometry optimization and frequency calculation [43,44,45,46,47,48]. Non-specific solvation was introduced by using the SMD continuum model [49] (toluene).

## 5. Conclusions

Dicyclopentylzinc readily reduces aldehydes to primary alcohols. The reaction with ketones has a less broad scope and is effective only with some heterocyclic ketones. The reaction is a synchronous transformation proceeding through a six-membered transition state. The use of a chiral catalyst, viz., (–)-(1*R*,2*S*)-ephedrine (**21**), leads to enantioselective reduction for 3-acetylpyridines **10** and **11**. Asymmetric amplifying autocatalysis occurs in the reduction of 3-acetylpyridine (**10**) catalyzed by its optically active product (*S*)-1-(pyridin-3-yl)ethanol (**19**).

## Figures and Tables

**Table 1:** Reduction of aldehydes with dicyclopentylzinc to the corresponding primary alcohols.

Entry	Starting Compound	Reaction Conditions	Product	Yield, % *^a^*
1	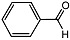	1 eq. ZnCp_2_, rt, 30 min, hexane	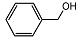	99
2		0.5 eq. ZnCp_2_, rt, 30 min, hexane		90
3	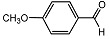	1 eq. ZnCp_2_, rt, 18 h, hexane	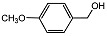	80
4	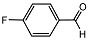	1 eq. ZnCp_2_, rt, 1 h, hexane	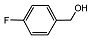	90
5	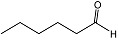	1 eq. ZnCp_2_, rt,1 h, hexane	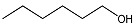	58
6	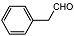	1 eq. ZnCp_2_, rt, 3.5 h, hexane	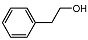	70
7	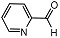	1 eq. ZnCp_2_, rt, 0.5 h, hexane	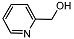	93
8	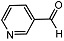	1 eq. ZnCp_2_, rt, 0.5 h, hexane	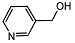	84
9	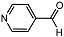	1 eq. ZnCp_2_, rt, 0.5 h, hexane	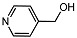	99

***^a^*** Yields were not optimized.

**Table 2:** Reduction of 3-acetylpyridines **10** and **11** with dicyclopentylzinc to the corresponding secondary alcohols **19** and **20**.

Entry	Starting Compound	Reaction Conditions	Product	Yield, % *^a^*	ee, % (HPLC)
1	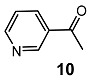	5 eq. ZnCp_2_, rt, 5 h, toluene	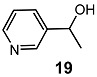	65	–
2		3 eq. ZnCp_2_, 0.1 eq. **21**, rt, 24 h		55	92 (*S*)
3		3 eq. ZnCp_2_, 0.1 eq. **21**, rt, 5 h		57	>99 (*S*)
4		3 eq. ZnCp_2_, 0.1 eq. (*S*)-**19**, *^b^* rt, 5 h		45 *^c^*^,*d*^	95 (*S*)
5		5 eq. ZnCp_2_, 0.1 eq. (*S*)-**19**, *^b^* rt, 5 h		42 *^c^*^,*d*^	96 (*S*)
6	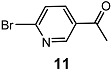	3 eq. ZnCp_2_, rt, 24 h, toluene	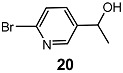	81	–
7		3 eq. ZnCp_2_, 0.1 eq. **21**, rt, 24 h		64	20

*^a^* Yields were not optimized; *^b^* alcohol **19**, 92% ee; *^c^* yield of the newly formed product; *^d^* yield of the product purified for HPLC, the NMR of the reaction mixture contained only **10** and **19**.

## Data Availability

The datasets used and/or analyzed during the current study are available from the corresponding author on reasonable request.

## Supplementary Materials

The following supporting information can be downloaded at: https://www.mdpi.com/article/10.3390/ijms242317048/s1 [23,24,25,26,27,28,29,30,31,32,33,34,35,36,37,38,39].

## References

[1] Soai K., Shibata T., Morioka H., Choji K. (1995). Asymmetric autocatalysis and amplification of enantiomeric excess of a chiral molecule. Nature.

[2] Soai K., Shibata T., Sato I. (2004). Discovery and development of asymmetric autocatalysis. Bull. Chem. Soc. Jpn..

[3] Mislow K. (2003). Absolute asymmetric synthesis: A commentary. Collect. Czech. Chem. Commun..

[4] Soai K., Sato I., Shibata T., Komiya S., Hayashi M., Matsueda Y., Imamura H., Hayase T., Morioka H., Tabira H. (2003). Asymmetric synthesis of pyrimidyl alkanol without adding chiral substances by the addition of diisopropylzinc to pyrimidine-5-carbaldehyde in conjunction with asymmetric autocatalysis. Tetrahedron Asymmetry.

[5] Gridnev I.D., Serafimov J.M., Quiney H., Brown J.M. (2003). Reflections on spontaneous asymmetric synthesis by amplifying autocatalysis. Org. Biomol. Chem..

[6] Singleton D.A., Vo L.K. (2003). A few molecules can control the enantiomeric outcome. Evidence supporting absolute asymmetric synthesis using the Soai asymmetric autocatalysis. Org. Lett..

[7] Tanji S., Kodaka Y., Ohno A., Shibata T., Sato I., Soai K. (2000). Asymmetric autocatalysis of 5-carbamoyl-3-pyridyl alkanols with amplification of enantiomeric excess. Tetrahedron Asymmetry.

[8] Athavale S.V., Simon A., Houk K.N., Denmark S.E. (2020). Structural contributions to autocatalysis and asymmetric amplification in the Soai reaction. J. Am. Chem. Soc..

[9] Lu N., Dai Z., Zhang Q., Zhang C., Kwong C.Y.R., Xia C. (2020). Metal Complex Containing Three Different. Ligands. Patent No..

[10] Chikugi Y., Komiya M., Furuta T., Iwamoto K., Takahashi Y., Nonoyama A. (1991). Preparation of New Benzoxazolone. Compounds. Patent No..

[11] Campbell J.B., Firor J.W. (2012). Synthesis of 5-substituted aminopyrrolo[3,4-b]quinolinones by a diorganozinc-mediated tandem alkylation/cyclization. Synth. Commun..

[12] Hyodo I., Tobisu M., Chatani N. (2012). Regioselective C–H bond functionalizations of acridines using organozinc reagents. Chem. Commun..

[13] Jadhav A.S., Anand R.V. (2017). 1,6-Conjugate addition of zinc alkyls to para-quinone methides in a continuous-flow microreactor. Org. Biomol. Chem..

[14] Angelini L., Sanz L.M., Leonori D. (2020). Divergent nickel-catalysed ring-opening–functionalization of cyclobutanone oximes with organozincs. Synlett.

[15] Schuppe A.W., Huang D., Chen Y., Newhouse T.R. (2018). Total synthesis of (−)-Xylogranatopyridine B via a palladium-catalyzed oxidative stannylation of enones. J. Am. Chem. Soc..

[16] Hatano M., Mizuno T., Ishihara K. (2010). Catalytic enantioselective synthesis of sterically demanding alcohols using di(2-alkyl)zinc prepared by the refined Charette’s method. Chem. Commun..

[17] Ishihara K., Hatano M. (2011). Preparation of Optically Active Alcohols. Patent No..

[18] Soai K., Niwa S., Kobayashi T. (1987). Asymmetric synthesis of pyridylethanols and amino alcohols by enantioselective reduction with lithium borohydride modified with N,N′-dibenzoylcystine. J. Chem. Soc. Chem. Commun..

[19] Tuulmets A., Sassian M. (1999). Reactions of partially solvated Grignard reagents with a ketone. J. Organomet. Chem..

[20] Bartolo N.D., Read J.A., Valentín E.M., Woerpel K.A. (2020). Reactions of allylmagnesium reagents with carbonyl compounds and compounds with C=N double bonds: Their diastereoselectivities generally cannot be analyzed using the Felkin–Anh and chelation-control models. Chem. Rev..

[21] Soai K., Niwa S., Hori H. (1990). Asymmetric self-catalytic reaction. Self-production of chiral 1-(3-pyridyl)alkanols as chiral self-catalysts in the enantioselective addition of dialkylzinc reagents to pyridine-3-carbaldehyde. J. Chem. Soc. Chem. Commun..

[22] Gridnev I.D., Vorobiev A.K. (2012). Quantification of sophisticated equilibria in the reaction pool and amplifying catalytic cycle of the Soai reaction. ACS Catal..

[23] Thiele K.H., Wilcke S., Ehrhardt M. (1968). Uber dicycloalkyl-verbindungen des zinks. J. Organomet. Chem..

[24] Integrated Spectral Data Base System of Organic Compounds, National Institute of Advanced Industrial Science and Technology (Japan). http://sdbs.db.aist.go.jp.

[25] Clarker M.L., Díaz-Valenzuela M.B., Slawin A.M.Z. (2007). Hydrogenation of aldehydes, esters, imines, and ketones catalyzed by a ruthenium complex of a chiral tridentate ligand. Organometallics.

[26] Jones G., Mouat D.J., Tonkinson D.J. (1985). Triazolopyridines. Part 6. Ring opening reactions of triazolopyridines. J. Chem. Soc. Perkin Trans..

[27] Cho S.-D., Park Y.-D., Kim J.-J., Falck J.R., Yoon Y.-J. (2004). Facile reduction of carboxylic acids, esters, acid chlorides, amides and nitriles to alcohols or amines using NaBH4/BF3⋅Et2O. Bull. Korean Chem. Soc..

[28] Basavaiah D., Sharada D.S., Veerendhar A. (2006). Organo-base mediated Cannizzaro reaction. Tetrahedron Lett..

[29] Knorr R., Lattke E. (1981). 2-Methyl-1-phenyl-1-propenyllithium. Ein katalytisch ummetallierbares Vinyllithiumderivat. Chem. Ber..

[30] Trecourt F., Breton G., Bonnet V., Mongin F., Marsais F., Queguiner G. (2000). New syntheses of substituted pyridines via bromine-magnesium exchange. Tetrahedron.

[31] Bolm C., Ewald M., Felder M., Schlingloff G. (1992). Enantioselective synthesis of optically active pyridine derivatives and C2-symmetric 2,2′-bipyridines. Chem. Ber..

[32] Lebedev Y., Polishchuk I., Maity B., Guerreiro M.D.V., Cavallo L., Rueping M. (2019). Asymmetric hydroboration of heteroaryl ketones by aluminumcatalysis. J. Am. Chem. Soc..

[33] Babaoglu K., Brizgys G., Cha J., Chen X., Guo H., Halcomb R.L., Han X., Huang R., Liu H., McFadden R. (2013). Benzothiazol-6-ylacetic acid derivatives as anti-HIV agents and their preparation and use for treating an HIV infection. Patent No..

[34] Subota A.I., Lutsenko A.O., Vashchenko B.V., Volochnyuk D.M., Levchenko V., Dmytriv Y.V., Rusanov E.B., Gorlova A.O., Ryabukhin S.V., Grygorenko O.O. (2019). Scalable and straightforward synthesis of all isomeric (cyclo)alkylpiperidines. Eur. J. Org. Chem..

[35] Gottarelli G., Samori B. (1974). Circular dichroism of isomeric pyridylethanols. J. Chem. Soc. Perkin Trans. 2.

[36] Hatano M., Suzuki S., Ishihara K. (2006). Highly efficient alkylation to ketones and aldimines with Grignard reagents catalyzed by Zinc(II) chloride. J. Am. Chem. Soc..

[37] Cook B.N., Disalvo D., Fandrick D.R., Harcken C., Kuzmich D., Lee T.W.H., Liu P., Lord J., Mao C., Neu J. (2010). Azaindazole compounds as ccr1 receptor antagonists. Patent No..

[38] Lunniss C.J., Palmer J.T., Pitt G.R.W., Axford L.C., Davies D. (2013). Antibacterial compounds. Patent No..

[39] Harriman G., Kolz C., Roth B., Song Y., Trivedi B. (2000). Functionalized heterocycles as chemokine receptor modulators. Patent No..

[40] Chai J.-D., Head-Gordon M. (2008). Long-range corrected hybrid density functionals with damped atom–atom dispersion corrections. Phys. Chem. Chem. Phys..

[41] Frisch M.J., Trucks G.W., Schlegel H.B., Scuseria G.E., Robb M.A., Cheeseman J.R., Scalmani G., Barone V., Petersson G.A., Nakatsuji H. (2016). Gaussian 16, Revision C.01.

[42] Becke A.D. (1988). Density-functional exchange-energy approximation with correct asymptotic behavior. Phys. Rev. A Gen. Phys..

[43] Ditchfield R., Hehre W.J., Pople J.A. (1971). Self-consistent molecular-orbital methods. IX. An extended Gaussian-type basis for molecular-orbital studies of organic molecules. J. Chem. Phys..

[44] Hehre W.J., Ditchfield R., Pople J.A. (1972). Self-consistent molecular orbital methods. XII. Further extensions of Gaussian-type basis sets for use in molecular orbital studies of organic molecules. J. Chem. Phys..

[45] Hariharan P.C., Pople J.A. (1973). The influence of polarization functions on molecular orbital hydrogenation energies. Theor. Chim. Acta.

[46] Francl M.M., Pietro W.J., Hehre W., Binkley J.S., Gordon M.S., DeFrees D.J., Pople J.A. (1982). Self-consistent molecular orbital methods. XXIII. A polarization-type basis set for second-row elements. J. Chem. Phys..

[47] Gordon M.S., Binkley J.S., Pople J.A., Pietro W.J., Hehre W.J. (1982). Self-consistent molecular-orbital methods. 22. Small split-valence basis sets for second-row elements. J. Am. Chem. Soc..

[48] Rassolov V.A., Pople J.A., Ratner M.A., Windus T.L. (1998). 6–31G* basis set for atoms K through Zn. J. Chem. Phys..

[49] Marenich A.V., Cramer C.J., Truhlar D.G. (2009). Universal solvation model based on solute electron density and on continuum model of the solvent defined by the bulk dielectric constant and atomic surface tensions. J. Phys. Chem. B.

