# Supplementary material for: The Reduction of Carbonyl Compounds with Dicyclopentylzinc: A New Example of Asymmetric Amplifying Autocatalysis
Source: Int J Mol Sci. 2023 Dec 1;24(23):17048. doi: 10.3390/ijms242317048 (PMC10707151; doi:10.3390/ijms242317048)
Supplement: Supplementary file 1 [file ijms-24-17048-s001.zip › ijms-2728831-supplementary.pdf]

Supplementary Materials

Reduction of Carbonyl Compounds with Dicyclopentylzinc: A New Example of Asymmetric Amplifying Autocatalysis

Elena Sh. Saigitbatalova [1]†, Liliya Z. Latypova [1]†, Almaz A. Zagidullin [2], Almira R. Kurbangalieva [1,*] and Ilya D. Gridnev [3,*]

[1] Biofunctional Chemistry Laboratory, A. Butlerov Institute of Chemistry, Kazan Federal University, 18 Kremlyovskaya Street, 420008 Kazan, Russia
[2] A. E. Arbuzov Institute of Organic and Physical Chemistry, FRC Kazan Scientific Center of RAS, 8 Arbuzov Street, 420088 Kazan, Russia
[3] N. D. Zelinsky Institute of Organic Chemistry, Leninsky Prosp. 47, 119991 Moscow, Russia

† These authors contributed equally to this work
* Correspondence: ilyaiochem@gmail.com (I.D.G.); akurbang@kpfu.ru (A.R.K.)

Content

1. Materials and Methods 3

2. Chemical Synthesis 4

2.1. Synthesis of dicyclopentylzinc 4

2.2. Reaction of aldehyde 1 with dicyclopentylzinc 4

2.3. General procedure for the reaction of aldehydes with dicyclopentylzinc 5

2.4. Reactions of aldehydes with diisopropylzinc 6

2.5. General procedure for the reaction of pyridine ketones with dicyclopentylzinc 6

2.6. General procedure for the reaction of pyridine ketones with dicyclopentylzinc in the presence of catalyst 8

2.7. Autocatalytic reaction of 3-acetylpyridine 10 with dicyclopentylzinc 9

2.8 The three-stage reaction sequence 10

3. References 12

4. HPLC data for alcohols 19 and 20 13

5. Cartesian coordinates 17

1. Materials and Methods

All reactions were carried out using standard Schlenk techniques under an argon atmosphere in oven-dried glassware with magnetic stirring. All solvents were purified and distilled using standard procedures. Solvents were additionally degassed by three pump–freeze–thaw cycles. Analytical thin-layer chromatography (TLC) was carried out on Sorbfil PTLC-AF-A-UF plates using UV light (254 nm) as the visualizing agent. Silica gel 60A (Acros Organics, 400–230 mesh, 0.040–0.063 mm) was used for open column chromatography. Melting points were recorded with a Boëtius melting point instrument and are uncorrected. NMR spectra were measured on a Bruker Avance III 400 spectrometer at 400.17 MHz (^{1}H) and 100.62 MHz (^{13}C), and a Bruker Avance III 500 spectrometer at 500.1 MHz (^{1}H) and 125.8 MHz (^{13}C) at 20 °C in the deuterated chloroform and benzene. The chemical shifts (δ) are expressed in parts per million (ppm) and are calibrated using residual undeuterated solvent peak as an internal reference ($CDCl_3$: δ_H 7.26, δ_C 77.16; C_6D_6: δ_H 7.16, δ_C 128.06). All coupling constants (*J*) are reported in Hertz (Hz), and multiplicities are indicated as follows: s (singlet), d (doublet), dd (doublet of doublets), qd (quartet of doublets) and m (multiplet). The enantiomeric excess (ee) measurements were performed via HPLC analysis on an HPLC system equipped with chiral stationary phase columns Chiralpak (OD-H), detection at 220 nm.

4-Methoxybenzaldehyde, 4-fluorobenzaldehyde, hexanal, 2-phenylacetaldehyde, pyridine-2-carbaldehyde, pyridine-3-carbaldehyde, pyridine-4-carbaldehyde, 2-acetylpyridine (**3**), 2-acetyl-6-bromopyridine (**4**), 2-acetyl-5-bromopyridine (**5**), 2-acetyl-4-bromopyridine (**6**), 2-acetyl-3-bromopyridine (**7**), 4-acetylpyridine (**8**), 4-acetyl-2-bromopyridine (**9**), 3-acetylpyridine (**10**), 5-acetyl-2-bromopyridine (**11**), bromocyclopentane, (–)-(1*R*,2*S*)-ephedrine (**21**) (Merck), benzaldehyde, magnesium (Acros), zinc chloride (TCI), Celite (Fluka), diisopropylzinc 1 M solution in toluene (Sigma-Aldrich) were used as received without further purification.

2. Chemical Synthesis

2-((Trimethylsilyl)ethynyl)pyrimidine-5-carbaldehyde (**1**) was synthesized according to the known method [1].

2.1. Synthesis of dicyclopentylzinc

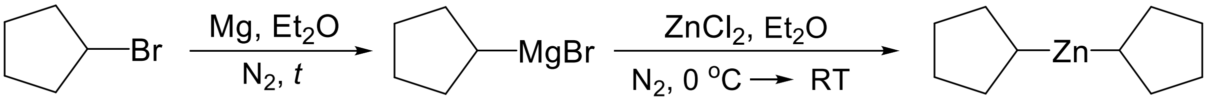

A solution of cyclopentyl bromide (7.2 mL, 67.1 mmol) in 10 mL of diethyl ether was added dropwise to a suspension of freshly calcined and iodine-activated magnesium (2.12 g, 87.2 mmol) in 20 mL of diethyl ether. The reaction mixture was refluxed for 2 h. $ZnCl_2$ (3.22 g, 23.6 mol) had been dried intensively in vacuo by flaming for 1 h. After cooling down to room temperature the solution of cyclopentylmagnesiumbromide obtained after filtration of the reaction mixture through the cannula was added to the suspension of $ZnCl_2$ in diethyl ether (5 mL) in an ice bath. The reaction mixture was stirred at room temperature for 12 h under nitrogen. Dry petroleum ether (30 mL) was added to the obtained suspension, and the resulting solution was filtered via the cannula to the round bottom Schlenck flask (this procedure was repeated twice). The volume of the filtrate was reduced in vacuo (1×10^{-3} mbar), and the remaining grey residue was distilled under reduced pressure (p = 3.7×10^{-3} mbar, bp = 65–67 °C (44–46 °C, p = 1×10^{-4} mm [2]) to yield the desired compound.

Dicyclopentylzinc: colorless solid, 3.23 g (67% yield); ^{1}H NMR (C_6D_6, 400 MHz, ppm) *δ* 1.95–1.77 (m, 4H, CH_2), 1.69–1.54 (m, 4H, CH_2), 1.53–1.33 (m, 8H, CH_2), 0.54–0.40 (m, 2H, CH); ^{13}C {^{1}H} NMR ($CDCl_3$, 125 MHz, ppm) *δ* 30.43 (CH_2), 29.64 (CH), 26.56 (CH_2).

For subsequent reactions, 10^{-3} M solutions of dicyclopentylzinc in dry hexane (for all aldehydes, except aldehyde **1**) or in dry toluene (for all ketones) were prepared.

*2.2. Reaction of aldehyde **1** with dicyclopentylzinc*

(2-((Trimethylsilyl)ethynyl)pyrimidin-5-yl)methanol (2). The solution of aldehyde **1** (0.10 g, 0.50 mmol) in dry toluene (3 mL) with intense stirring was added to the mixture of 10^{-3} M solution of dicyclopentylzinc (1.0 mL) with 6 mL of dry toluene under nitrogen atmosphere at –70 °C (acetone/dry ice bath). The reaction mixture was stirred for 30 min at –70 °C and for 3 h at room temperature. The mixture was then quenched with aqueous HCl solution (1 M, 5 mL). After stirring for 15 min, the reaction mixture was neutralized with a saturated solution of $NaHCO_3$ (7 mL). The organic phase was separated, and the aqueous layer was extracted with diethyl ether (4 × 15 mL). The combined organic layers were dried over Na_2CO_3, and the solvent was removed in vacuo. The remaining orange solid was purified via column chromatography (eluent petroleum ether–acetone 8:1) to yield alcohol **2**. Spectral data are consistent with the literature [1].

2.3. General procedure for the reaction of aldehydes with dicyclopentylzinc

The solution of aldehyde (0.50 mmol) in dry hexane (3 mL) with intense stirring was added to the mixture of 10^{-3} M solution of dicyclopentylzinc with 7 mL of dry hexane under a nitrogen atmosphere. The reaction mixture was stirred at room temperature and was then quenched with aqueous HCl solution (1 M, 3 mL). After stirring for 15 min, the reaction mixture was neutralized with a saturated solution of $NaHCO_3$ (5 mL). The organic phase was separated, and the aqueous layer was extracted with diethyl ether (3 × 20 mL). The combined organic layer was filtered through the Celite and removed in vacuo. The residue was analyzed via NMR spectroscopy without additional purification.

Benzyl alcohol was obtained according to the general procedure from benzaldehyde (51 μL, 0.50 mmol) and dicyclopentylzinc (10^{-3} M solution, 0.50 mL). The reaction time was 30 min. The yield was quantitative. Spectral data are consistent with the database [3].

(4-Methoxyphenyl)methanol was obtained according to the general procedure from 4-methoxybenzaldehyde (61 μL, 0.50 mmol) and dicyclopentylzinc (10^{-3} M solution, 0.50 mL). The reaction time was 18 h. The yield (80%) was determined according to ^{1}H NMR spectra of the crude product. Spectral data are consistent with the database [3].

(4-Fluorophenyl)methanol was obtained according to the general procedure from 4-fluorobenzaldehyde (53 μL, 0.50 mmol) and dicyclopentylzinc (10^{-3} M solution, 0.50 mL). The reaction time was 1 h. Light yellow oil was obtained, 57 mg (90% yield). Spectral data are consistent with the literature [4].

Hexanol-1 was obtained according to the general procedure from hexanal (62 μL, 0.50 mmol) and dicyclopentylzinc (10^{-3} M solution, 0.50 mL). The reaction time was 1 h. The yield (58%) was determined according to ^{1}H NMR spectra of the crude product. Spectral data are consistent with the database [3].

2-Phenylethanol was obtained according to the general procedure from 2-phenylacetaldehyde (56 μL, 0.50 mmol) and dicyclopentylzinc (10^{-3} M solution, 0.50 mL). The reaction time was 3.5 h. The yield (70%) was determined according to ^{1}H NMR spectra of the crude product. Spectral data are consistent with the database [3].

Pyridin-2-ylmethanol was obtained according to the general procedure from pyridine-2-carbaldehyde (48 μL, 0.50 mmol) and dicyclopentylzinc (10^{-3} M solution, 0.50 mL). The reaction time was 30 min. The residue obtained after evaporation of the solvents was purified via column chromatography (eluent ethyl acetate–petroleum ether, 1:1). Colorless oil was obtained, 51 mg (93% yield); R_f 0.29 (ethyl acetate–petroleum ether, 1:1). Spectral data are consistent with the literature [5].

Pyridin-3-ylmethanol was obtained according to the general procedure from pyridine-3-carbaldehyde (47 μL, 0.50 mmol) and dicyclopentylzinc (10^{-3} M solution, 0.50 mL). The reaction time was 30 min. The residue obtained after evaporation of the solvents was purified via column chromatography (eluent ethyl acetate–petroleum ether, 1:1). Colorless oil, 46 mg (84% yield); R_f 0.31 (ethyl acetate–petroleum ether, 1:1). Spectral data are consistent with the literature [6].

Pyridin-4-ylmethanol was obtained according to the general procedure from pyridine-4-carbaldehyde (47 μL, 0.50 mmol) and dicyclopentylzinc (10^{-3} M solution, 0.50 mL). The reaction time was 30 min. The residue obtained after evaporation of the solvents was purified via column chromatography (eluent ethyl acetate–petroleum ether, 1:2). Colorless solid, 54 mg (99%

yield); mp 58–59 °C (55–57 °C [7]); R_f 0.58 (ethyl acetate–petroleum ether, 1:2). Spectral data are consistent with the literature [7].

2.4. Reactions of aldehydes with diisopropylzinc

To the solution of hexanal (62 μL, 0.50 mmol) in dry hexane (5 mL), a toluene solution of $(^iPr)_2Zn$ (1 M, 0.5 mL) was added dropwise via syringe with intense stirring under an argon atmosphere. The reaction mixture was stirred for 3 days at room temperature and was then quenched with aqueous HCl solution (0.1 M, 5 mL). After stirring for 15 min, the reaction mixture was neutralized with a saturated solution of $NaHCO_3$ (7 mL). The organic phase was separated, and the aqueous layer was extracted with CH_2Cl_2 (3 × 10 mL). The solvent from the combined organic layers was removed in vacuo. According to the HPLC and GC–MS spectroscopy, the remaining residue contained 2-methyloctan-3-ol (9%) with other byproducts.

The reaction of benzaldehyde (51 μL, 0.50 mmol) with $(^iPr)_2Zn$ (1 M, 0.5 mL) was carried out as described above for hexanal. The reaction time was 26 h. The residue obtained after evaporation of the solvents was purified via column chromatography (eluent ethyl acetate–petroleum ether, 1:6). The fraction with R_f 0.86 contained starting aldehyde (37 mg, 69%), and the fraction with R_f 0.75 contained **2-methyl-1-phenylpropan-1-ol**. Colorless oil was obtained, 20 mg (26% yield). Spectral data are consistent with the literature [8].

2.5. General procedure for the reaction of pyridine ketones with dicyclopentylzinc

The solution of ketone (0.45 mmol) in dry toluene (3 mL) with intense stirring was added to the mixture of 10^{-3} M solution of dicyclopentylzinc (2.3 mL) with 7 mL of dry toluene under a nitrogen atmosphere. The reaction mixture was stirred for 5 or 24 h at room temperature and was then quenched with aqueous HCl solution (1 M, 5 mL). After stirring for 15 min, the reaction mixture was neutralized with a saturated solution of $NaHCO_3$ (7 mL). The organic phase was separated, and the aqueous layer was extracted with ethyl acetate (3 × 20 mL). The combined organic layer was filtered through the Celite and removed in vacuo. The remaining yellow oil was purified via column chromatography (eluent acetone–toluene) to yield the corresponding alcohol.

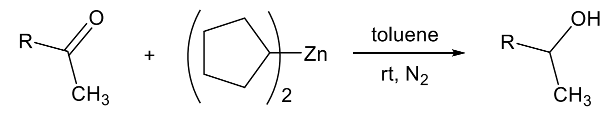

3 - 11 **12 - 20**

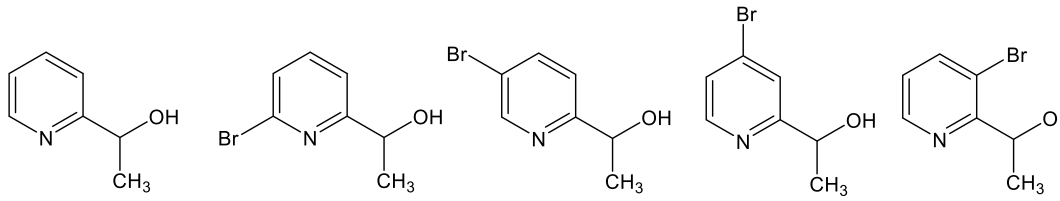

12 (67%) **13** (63%) **14** (76%) **15** (84%) **16** (63%)

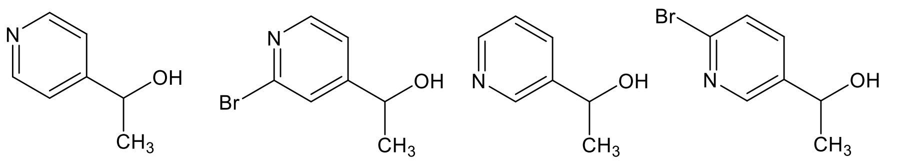

17 (67%) **18** (86%) **19** (65%) **20** (81%)

1-(Pyridin-2-yl)ethanol (12) was synthesized according to the general procedure from 2-acetylpyridine (**3**, 0.05 mL, 0.45 mmol) and dicyclopentylzinc (10^{-3} M solution, 2.25 mL). The reaction time was 5 h. Eluent for the column chromatography: acetone–toluene, from 1:2 to 1:1. Colorless solid, 37 mg (67% yield); mp 37–39 °C (38–40 °C [9]); R_f 0.16 (acetone–toluene, 1:2); ^{1}H NMR ($CDCl_3$, 400 MHz, ppm) δ 8.54 (d, 1H, $H_{arom.}$), 7.75–7.62 (m, 1H, $H_{arom.}$), 7.33–7.15 (m, 2H, $H_{arom.}$), 4.89 (q, 1H, <u>CH</u>-OH, ^3J = 6.5 Hz), 4.26 (br s, 1H, OH), 1.50 (d, 3H, CH_3, ^3J = 6.5 Hz). Spectral data are consistent with match the literature [9].

1-(6-Bromopyridin-2-yl)ethanol (13) was synthesized according to the general procedure from 2-acetyl-6-bromopyridine (**4**, 0.09 g, 0.45 mmol) and dicyclopentylzinc (10^{-3} M solution, 1.34 mL). The reaction time was 5 h. Eluent for the column chromatography: acetone–toluene, from 1:20 to 1:3. Light yellow oil, 57 mg (63% yield); R_f 0.31 (acetone–toluene, 1:10). Spectral data are consistent with the literature [10].

1-(5-Bromopyridin-2-yl)ethanol (14) was synthesized according to the general procedure from 2-acetyl-5-bromopyridine (**5**, 0.09 g, 0.45 mmol) and dicyclopentylzinc (10^{-3} M solution, 1.34 mL). The reaction time was 24 h. Eluent for the column chromatography: acetone–toluene, from 1:10 to 1:6. Colorless solid, 69 mg (76% yield); mp 77–80 °C; R_f 0.32 (acetone–toluene, 1:6). Spectral data are consistent with the literature [11].

1-(4-Bromopyridin-2-yl)ethanol (15) was synthesized according to the general procedure from 2-acetyl-4-bromopyridine (**6**, 0.05 g, 0.25 mmol) and dicyclopentylzinc (10^{-3} M solution, 0.75 mL). The reaction time was 5 h. According to the ^{1}H NMR spectroscopy, the reaction mixture contained 10% of starting ketone **6**. Eluent for the column chromatography: acetone–toluene, from 1:10 to 1:5. Colorless crystals, 43 mg (84% yield); mp 67–69 °C; R_f 0.24 (acetone–toluene, 1:5). Spectral data are consistent with the literature [12].

1-(3-Bromopyridin-2-yl)ethanol (16) was synthesized according to the general procedure from 2-acetyl-3-bromopyridine (**7**, 0.09 g, 0.45 mmol) and dicyclopentylzinc (10^{-3} M solution, 1.34 mL). The reaction time was 24 h. Eluent for the column chromatography: acetone–toluene, 1:10. Colorless oil, 57 mg (63% yield); R_f 0.34 (acetone–toluene, 1:10). Spectral data are consistent with the literature [13].

1-(Pyridin-4-yl)ethanol (17) was synthesized according to the general procedure from 4-acetylpyridine (**8**, 0.05 mL, 0.45 mmol) and dicyclopentylzinc (10^{-3} M solution, 1.35 mL). The

reaction time was 5 h. Eluent for the column chromatography: acetone–toluene, from 1:10 to 1:1. Colorless solid, 37 mg (67% yield); mp 53–54.5 °C (57–59 °C [14]; R_f 0.31 (acetone–toluene, 1:1). Spectral data are consistent with the literature [15].

1-(2-Bromopyridin-4-yl)ethanol (18) was synthesized according to the general procedure from 4-acetyl-2-bromopyridine (**9**, 0.05 g, 0.25 mmol) and dicyclopentylzinc (10^{-3} M solution, 0.75 mL). The reaction time was 24 h. According to the ^{1}H NMR spectroscopy, the reaction mixture contained 7% of starting ketone **9**. Eluent for the column chromatography: acetone–toluene, 1:20. Colorless oil, 43 mg (86% yield); R_f 0.23 (acetone–toluene, 1:20); ^{1}H NMR ($CDCl_3$, 400 MHz, ppm) δ 8.29 (d, 1H, H_{arom}, ^3J = 5.1 Hz), 7.52 (br s, 1H, H_{arom}), 7.24 (dd, 1H, H_{arom}, ^3J = 5.1 Hz, ^4J = 1.1 Hz), 4.88 (qd, 1H, <u>CH</u>–OH, ^3J = 6.6 Hz, ^3J = 3.8 Hz), 2.36 (d, 1H, OH, ^3J = 3.8 Hz), 1.48 (d, 3H, CH_3, ^3J = 6.6 Hz); $^{13}C\{^1H\}$ NMR ($CDCl_3$, 100 MHz, ppm) δ 157.81, 150.34, 142.69, 124.88, 119.73 (C_{arom}), 68.59 (CH), 25.26 (CH_3). Alcohol **18** is known [16, 17]; however, no spectroscopic data are available.

1-(Pyridin-3-yl)ethanol (19) was synthesized according to the general procedure from 3-acetylpyridine (**10**, 0.05 mL, 0.45 mmol) and dicyclopentylzinc (10^{-3} M solution, 2.27 mL). The reaction time was 5 h. According to the ^{1}H NMR spectroscopy, the reaction mixture contained 10% of starting ketone **10**. Eluent for the column chromatography: acetone–petroleum ether, from 1:2 to 1:1. Colorless oil, 36 mg (65% yield); R_f 0.31 (acetone–petroleum ether, 1:1). Spectral data are consistent with the literature [9].

1-(6-Bromopyridin-3-yl)ethanol (20) was synthesized according to the general procedure from 5-acetyl-2-bromopyridine (**11**, 0.09 g, 0.45 mmol) and dicyclopentylzinc (10^{-3} M solution, 1.34 mL). The reaction time was 24 h. According to the ^{1}H NMR spectroscopy, the reaction mixture contained 8% of starting ketone **11**. Eluent for the column chromatography: acetone–toluene, from 1:10 to 1:2. Colorless solid, 73 mg (81% yield); mp 47–49 °C (47–48 °C [10]); R_f 0.41 (acetone–toluene, 1:2). Spectral data are consistent with the literature [10].

2.6. General procedure for the reaction of pyridine ketones with dicyclopentylzinc in the presence of catalyst

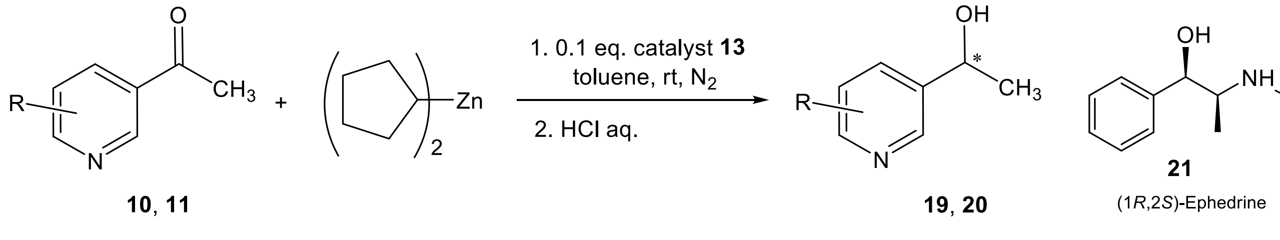

To the solution of dicyclopentylzinc (3 or 5 eq.) in dry toluene (20 mL), (–)-(1*R*,2*S*)-ephedrine (**21**) (0.1 eq.) was added under a nitrogen atmosphere. After stirring the mixture for 20 min at room temperature, a solution of ketone (1 eq.) in dry toluene (9 mL) was added dropwise. The reaction mixture was stirred for 5 or 24 h at room temperature and was then quenched with aqueous HCl solution (1 M, 5 mL). After stirring for 15 min, the reaction mixture was neutralized with a saturated solution of $NaHCO_3$ (7 mL). The organic phase was separated, and the aqueous layer was extracted with ethyl acetate (3 × 20 mL). The combined organic layer was filtered through the Celite and removed in vacuo. The remaining yellow oil was purified via column chromatography to yield the corresponding alcohol. The determination of the ee values of the isolated samples was performed via chiral HPLC.

Experiment № 1. Alcohol 19 was synthesized according to the general procedure from ketone **10** (0.15 mL, 1.34 mmol), catalyst **21** (22 mg, 0.13 mmol), and dicyclopentylzinc (10^{-3} M solution, 4.0 mL). The reaction time was 24 h. According to the ^{1}H NMR spectroscopy, the reaction mixture contained 13% of starting ketone **10**. Eluent for the column chromatography: acetone–petroleum ether, from 1:2 to 1:1. Colorless oil, 90 mg (55% yield); 92% ee (Chiralpak, OD, 2.0 mm × 60 mm, *i*PrOH / hexane = 10 / 90, 1.0 mL/min, 20 °C, 220 nm UV detector, retention time = 8.49 min for the (*R*) isomer and 9.68 min for the (*S*) isomer).

Experiment № 2. Alcohol 19 was synthesized according to the general procedure from ketone **10** (0.70 mL, 6.37 mmol), catalyst **21** (105 mg, 0.64 mmol), and dicyclopentylzinc (10^{-3} M solution, 19.1 mL). The reaction time was 5 h. Colorless oil, 0.45 g (57% yield); 99% ee (Chiralpak, OD, 2.0 mm × 60 mm, *i*PrOH / hexane = 10 / 90, 1.0 mL/min, 20 °C, 220 nm UV detector, retention time = 7.78 min for the (*R*) isomer and 9.77 min for the (*S*) isomer).

Alcohol 20 was synthesized according to the general procedure from ketone **11** (0.27 g, 1.34 mmol), catalyst **21** (22 mg, 0.13 mmol), and dicyclopentylzinc (10^{-3} M solution, 4.0 mL). The reaction time was 24 h. According to the ^{1}H NMR spectroscopy, the reaction mixture contained 6% of starting ketone **11**. Eluent for the column chromatography: acetone–toluene, from 1:10 to 1:8. Colorless solid, 173 mg (64% yield); ee 20% (Chiralpak, OD, 2.0 mm × 60 mm, *i*PrOH / hexane = 10 / 90, 1.0 mL/min, 20 °C, 220 nm UV detector, retention time = 8.17 min for the (*R*) isomer and 9.60 min for the (*S*) isomer).

2.7. Autocatalytic reaction of 3-acetylpyridine ***10*** *with dicyclopentylzinc*

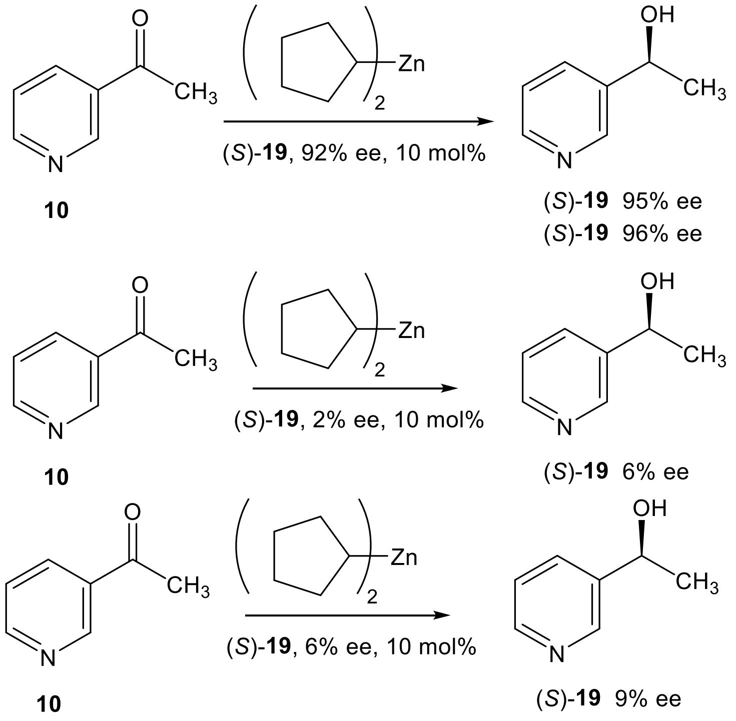

Experiment № 1: To the solution of alcohol (*S*)-**19** (17 mg, 0.14 mmol, 92% ee) in dry toluene (20 mL), the solution of dicyclopentylzinc (10^{-3} M solution, 4.1 mL) was added dropwise with intense stirring under a nitrogen atmosphere. The obtained mixture was stirred for 20 min at room temperature. Ketone **10** (0.15 mL, 1.38 mmol) was dissolved in 9 mL of dry toluene and

then was added dropwise to the reaction mixture under a nitrogen atmosphere. The reaction mixture was stirred for 5 h at room temperature. Workup of the reaction mixture was carried out similarly to the general procedure described above. According to the ^{1}H NMR spectroscopy, the reaction mixture contained 30% of starting ketone **10**. The yellow oily residue was purified via column chromatography (eluent acetone–petroleum ether, from 1:2 to 1:1) to yield the corresponding alcohol **19**. Colorless oil, 88 mg (42% yield), 95% ee (Chiralpak, OD, 2.0 mm × 60 mm, *i*PrOH / hexane = 10 / 90, 1.0 mL/min, 20 °C, 220 nm UV detector, retention time = 7.93 min for the (*R*) isomer and 9.29 min for the (*S*) isomer).

Experiment № 2: Reaction conditions were identical to those described above. Alcohol **19** was obtained from ketone **10** (0.14 mL, 1.29 mmol), dicyclopentylzinc (10^{-3} M solution, 6.5 mL), and alcohol (*S*)-**19** (16 mg, 0.13 mmol, 92% ee). Colorless oil, 87 mg (45% yield), 96% ee (Chiralpak, OD, 2.0 mm × 60 mm, *i*PrOH / hexane = 10 / 90, 1.0 mL/min, 20 °C, 220 nm UV detector, retention time = 8.26 min for the (*R*) isomer and 9.47 min for the (*S*) isomer).

Experiment № 3 (2% ee of catalyst): Reaction conditions were identical to those described above. Alcohol **19** was obtained from ketone **10** (0.14 mL, 1.27 mmol), dicyclopentylzinc (10^{-3} M solution, 3.8 mL), and alcohol **19** (16 mg, 0.13 mmol, 2% ee*). Colorless oil, 106 mg (58% yield); 6% ee (Chiralpak, OD, 2.0 mm × 60 mm, *i*PrOH / hexane = 10 / 90, 1.0 mL/min, 20 °C, 220 nm UV detector, retention time = 8.74 min for the (*R*) isomer and 9.41 min for the (*S*) isomer).

Experiment № 4 (6% ee of catalyst): Reaction conditions were identical to those described above. Alcohol **19** was obtained from ketone **10** (0.13 mL, 1.15 mmol), dicyclopentylzinc (10^{-3} M solution, 3.5 mL), and alcohol **19** (14 mg, 0.115 mmol, 6% ee*). The reaction mixture was stirred for 24 h at room temperature. Colorless oil, 100 mg (60% yield), 9% ee (Chiralpak, OD, 2.0 mm × 60 mm, *i*PrOH / hexane = 10 / 90, 1.0 mL/min, 20 °C, 220 nm UV detector, retention time = 8.41 min for the (*R*) isomer and 9.04 min for the (*S*) isomer).

Note: *ee was achieved by mixing racemic alcohol **19** with the sample, obtained from experiment № 2 in section 2.6 of Supplementary materials. Racemic alcohol **19** was obtained via the reduction of ketone **10** with $NaBH_4$ [18].

2.8 The three-stage reaction sequence

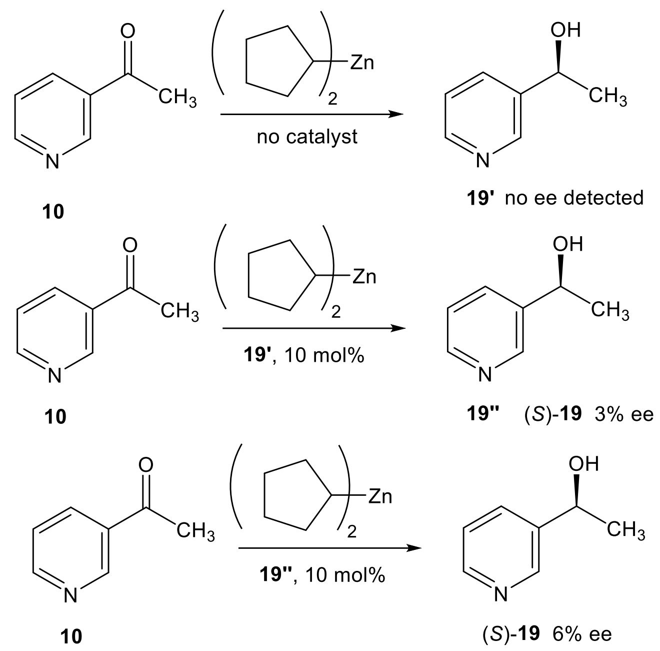

Stage 2. Reaction conditions were identical to those described above. Alcohol **19''** was obtained from ketone **10** (0.14 mL, 1.29 mmol), dicyclopentylzinc (10^{-3} M solution, 3.9 mL), and alcohol **19'** (16 mg, 0.13 mmol, 0% ee), obtained from the noncatalytic experiment described in section 2.5 of Supplementary materials. Colorless oil, 116 mg (59% yield), 3% ee (Chiralpak, OD, 2.0 mm × 60 mm, *i*PrOH / hexane = 10 / 90, 1.0 mL/min, 20 °C, 220 nm UV detector, retention time = 8.70 min for the (*R*) isomer and 9.38 min for the (*S*) isomer).

Stage 3. Reaction conditions were identical to those described above. Alcohol **19** was obtained from ketone **10** (0.14 mL, 1.29 mmol), dicyclopentylzinc (10^{-3} M solution, 3.9 mL), and alcohol **19''** (16 mg, 0.13 mmol, 3% ee), obtained from **Stage 2**. Colorless oil, 120 mg (65% yield), 6% ee (Chiralpak, OD, 2.0 mm × 60 mm, *i*PrOH / hexane = 10 / 90, 1.0 mL/min, 20 °C, 220 nm UV detector, retention time = 8.71 min for the (*R*) isomer and 9.41 min for the (*S*) isomer).

3. References

1. Gridnev, I.D.; Serafimov, J.M.; Quiney, H.; Brown, J.M. Reflections on spontaneous asymmetric synthesis by amplifying autocatalysis. *Org. Biomol. Chem.* **2003**, *1*, 3811–3819.
2. Thiele, K.H.; Wilcke, S.; Ehrhardt, M. Uber Dicycloalkyl-verbindungen des zinks. *J. Organomet. Chem.* **1968**, *14*, 13–19.
3. Integrated spectral data base system of organic compounds, National institute of advanced industrial science and technology (Japan). Available at: http://sdbs.db.aist.go.jp (accessed 30 November 2023).
4. Clarker, M.L.; Díaz-Valenzuela, M.B.; Slawin, A.M.Z. Hydrogenation of aldehydes, esters, imines, and ketones catalyzed by a ruthenium complex of a chiral tridentate ligand. *Organometallics* **2007**, *26*, 16–19.
5. Jones, G.; Mouat, D.J.; Tonkinson, D.J. Triazolopyridines. Part 6. Ring opening reactions of triazolopyridines. *J. Chem. Soc., Perkin Trans.* **1985**, *1*, 2719–2723.
6. Cho, S.-D.; Park, Y.-D.; Kim, J.-J.; Falck, J.R.; Yoon, Y.-J. Facile reduction of carboxylic acids, esters, acid chlorides, amides and nitriles to alcohols or amines using $NaBH_4/BF_3{\cdot}Et_2O$. *Bull. Korean Chem. Soc.* **2004**, *25*, 407–409.
7. Basavaiah, D.; Sharada, D.S.; Veerendhar, A. Organo-base mediated Cannizzaro reaction. *Tetrahedron Lett.* **2006**, *47*, 5771–5774.
8. Knorr, R.; Lattke, E. 2-Methyl-1-phenyl-1-propenyllithium. Ein katalytisch ummetallierbares Vinyllithiumderivat. *Chem. Ber.* **1981**, *114*, 2116–2131.
9. Trecourt, F.; Breton, G.; Bonnet, V.; Mongin, F.; Marsais, F.; Queguiner, G. New syntheses of substituted pyridines via bromine-magnesium exchange. *Tetrahedron* **2000**, *56*, 1349–1360.
10. Bolm, C.; Ewald, M.; Felder, M.; Schlingloff, G. Enantioselective synthesis of optically active pyridine derivatives and C_2-symmetric 2,2′-bipyridines. *Chem. Ber.* **1992**, *125*, 1169–1190.
11. Lebedev, Y.; Polishchuk, I.; Maity, B.; Guerreiro, M.D.V.; Cavallo, L.; Rueping, M. Asymmetric hydroboration of heteroaryl ketones by aluminumcatalysis. *J. Am. Chem. Soc.* **2019**, *141*, 19415−19423.
12. Babaoglu, K; Brizgys, G.; Cha, J.; Chen, X.; Guo, H.; Halcomb, R.L.; Han, X.; Huang, R.; Liu, H.; McFadden, R.; Mitchell, M.L.; Qi, Y.; Roethle, P.A.; Xu, L.; Yang, H. Benzothiazol-6-ylacetic acid derivatives as anti-HIV agents and their preparation and use for treating an HIV infection. Pat. WO2013159064, Oct. 24, **2013**.
13. Subota, A.I.; Lutsenko, A.O.; Vashchenko, B.V.; Volochnyuk, D.M.; Levchenko, V.; Dmytriv, Y.V.; Rusanov, E.B.; Gorlova, A.O.; Ryabukhin, S.V.; Grygorenko, O.O. Scalable and straightforward synthesis of all isomeric (cyclo)alkylpiperidines. *Eur. J. Org. Chem.* **2019**, *22*, 3636–3648.
14. Gottarelli, G.; Samori, B. Circular dichroism of isomeric pyridylethanols. *J. Chem. Soc., Perkin Trans*. 2. **1974**, *12*, 1462–1465.
15. Hatano, M.; Suzuki, S.; Ishihara, K. Highly efficient alkylation to ketones and aldimines with Grignard reagents catalyzed by Zinc(II) chloride. *J. Am. Chem. Soc.* **2006**, *128*, 9998–9999.
16. Cook, B.N.; Disalvo, D.; Fandrick, D.R.; Harcken, C.; Kuzmich, D.; Lee, T.W.H.; Liu, P.; Lord, J.; Mao, C.; Neu, J.; Raudenbush, B.C.; Razavi, H.; Reeves, J.T.; Song, J.J.; Swinamer, A.D.; Tan, Z. Azaindazole compounds as ccr1 receptor antagonists.,Pat. WO2010036632A1, Apr. 1, **2010**.
17. Lunniss, C.J.; Palmer, J.T; Pitt, G.R.W.; Axford, L.C.; Davies, D. Antibacterial compounds. Pat. WO2013138860, Sept. 26, **2013**.
18. Harriman, G.; Kolz, C.; Roth, B.; Song, Y.; Trivedi, B. Functionalized heterocycles as chemokine receptor modulators. Pat. WO00/42045, July 20, **2000**.

4. HPLC data for alcohols 19 and 20

Conditions: *i*PrOH – hexane = 10 : 90
Flow rate = 1.0 mL/min, λ = 220 nm, Chiralpak OD

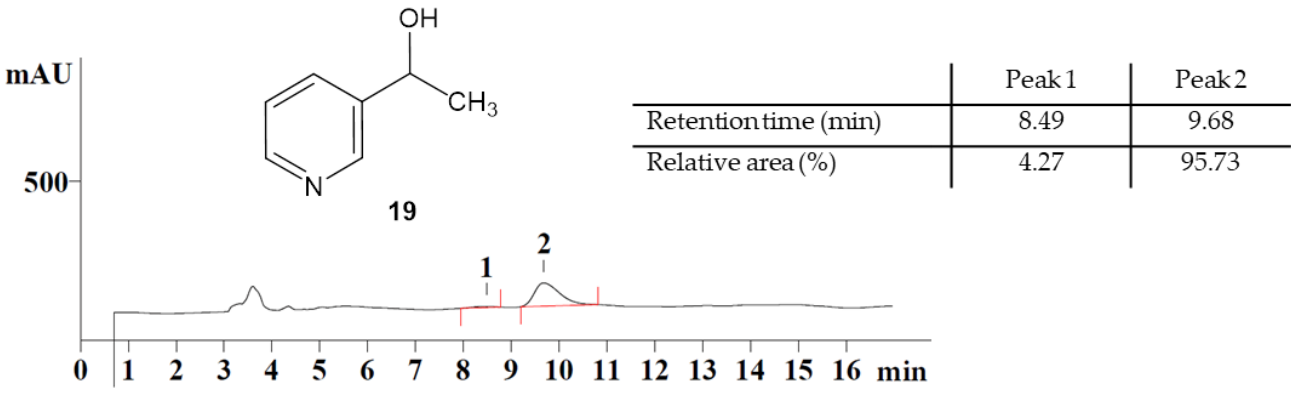

	Peak 1	Peak 2
Retention time (min)	8.49	9.68
Relative area (%)	4.27	95.73

Figure S1. HPLC chromatogram for compound **19**, obtained in the experiment № 1 with catalyst **21**.

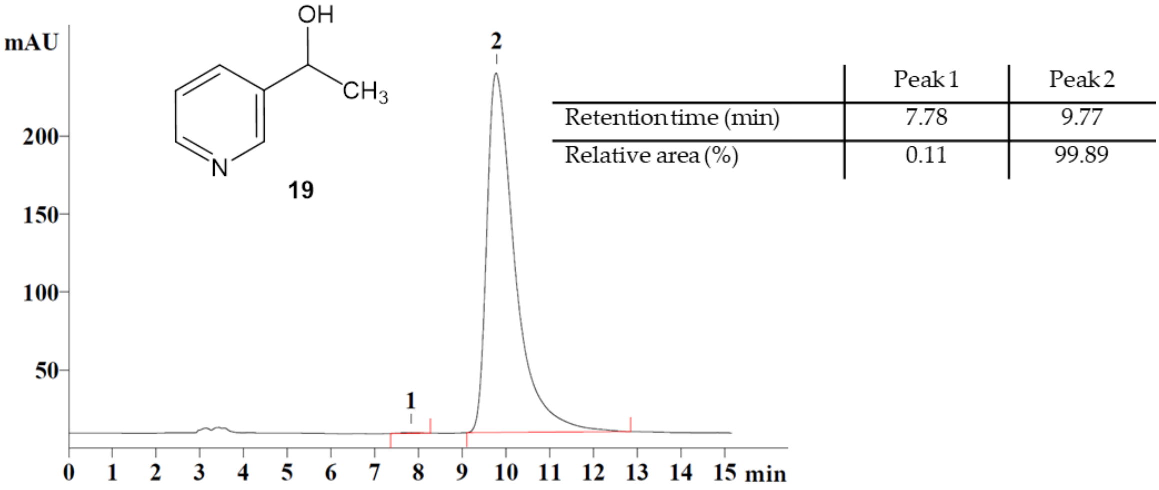

	Peak 1	Peak 2
Retention time (min)	7.78	9.77
Relative area (%)	0.11	99.89

Figure S2. HPLC chromatogram for compound **19**, obtained in the experiment № 2 with catalyst **21**.

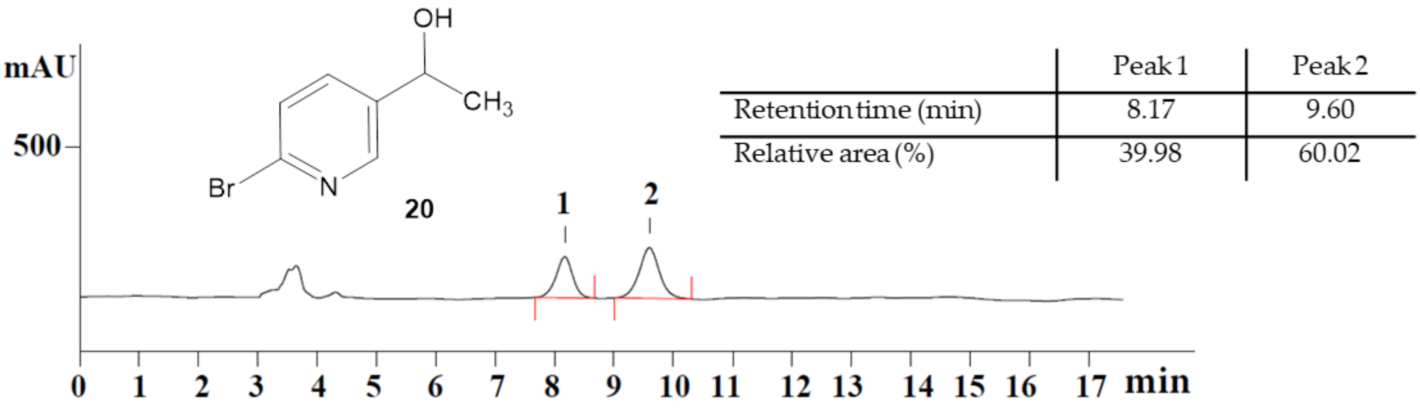

	Peak 1	Peak 2
Retention time (min)	8.17	9.60
Relative area (%)	39.98	60.02

Figure S3. HPLC chromatogram for compound **20**, obtained in the experiment with catalyst **21**.

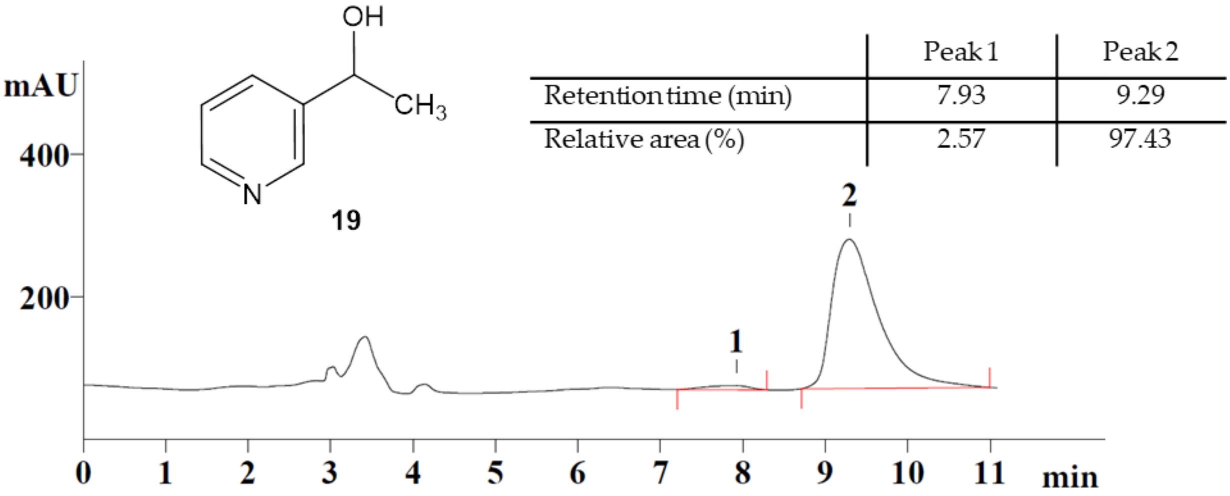

	Peak 1	Peak 2
Retention time (min)	7.93	9.29
Relative area (%)	2.57	97.43

Figure S4. HPLC chromatogram for compound **19**, obtained in the autocatalytic reaction (experiment № 1) with catalyst (*S*)-**19**.

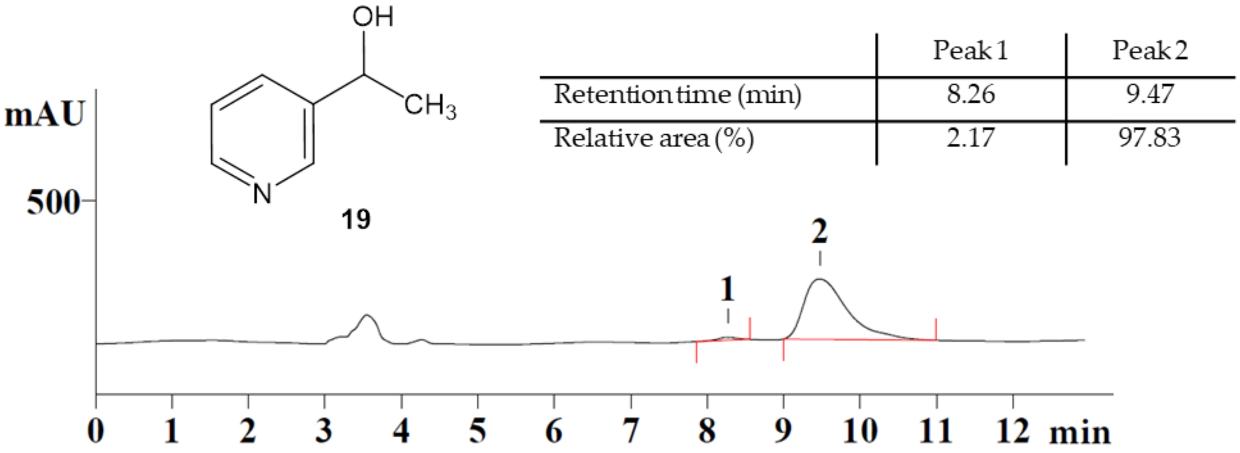

	Peak 1	Peak 2
Retention time (min)	8.26	9.47
Relative area (%)	2.17	97.83

Figure S5. HPLC chromatogram for compound **19**, obtained in the autocatalytic reaction (experiment № 2) with catalyst (*S*)-**19**.

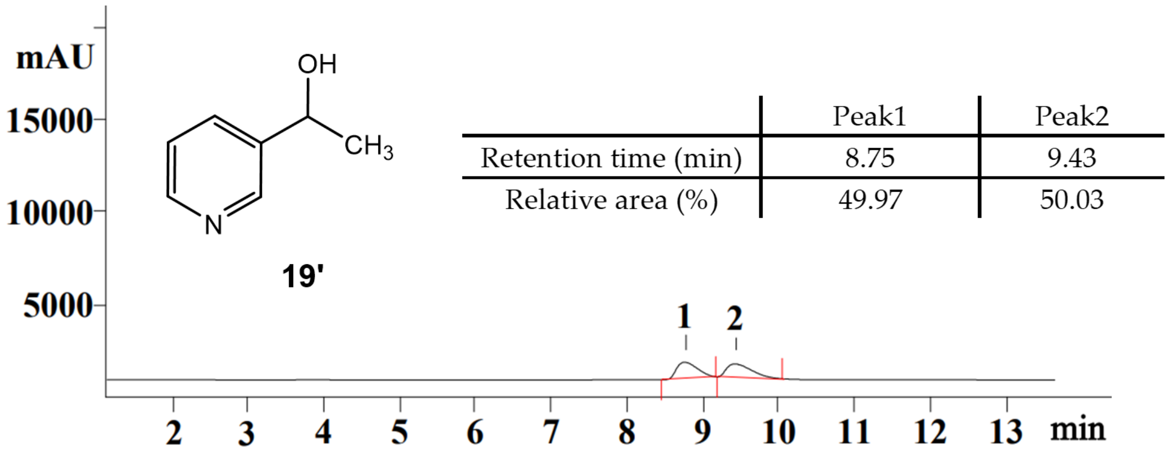

	Peak1	Peak2
Retention time (min)	8.75	9.43
Relative area (%)	49.97	50.03

Figure S6. HPLC chromatogram for the sample **19′**, obtained in the non-catalytic reaction of ketone **10** with dicyclopentylzinc.

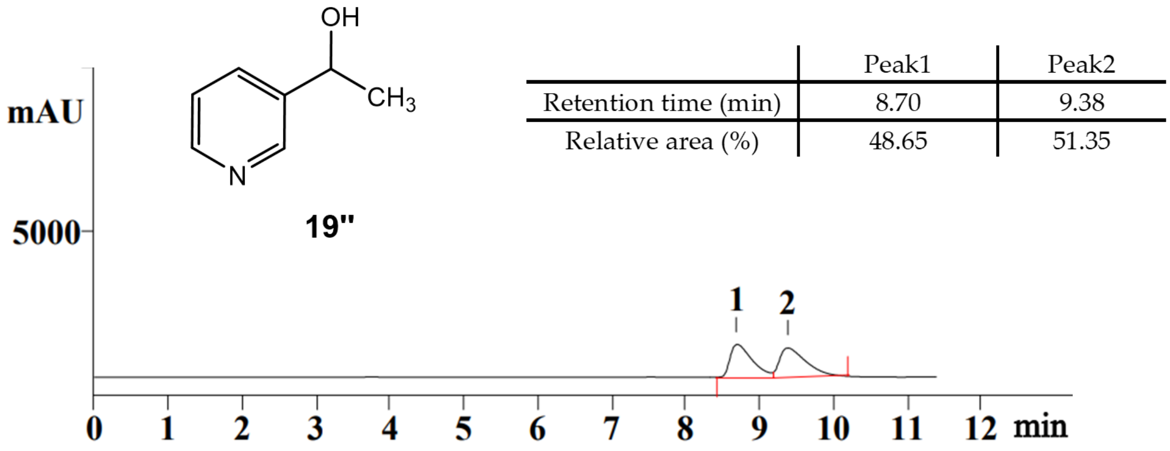

	Peak1	Peak2
Retention time (min)	8.70	9.38
Relative area (%)	48.65	51.35

Figure S7. HPLC chromatogram for the sample **19′′**, obtained in the autocatalytic reaction (stage 2) with catalyst **19′**.

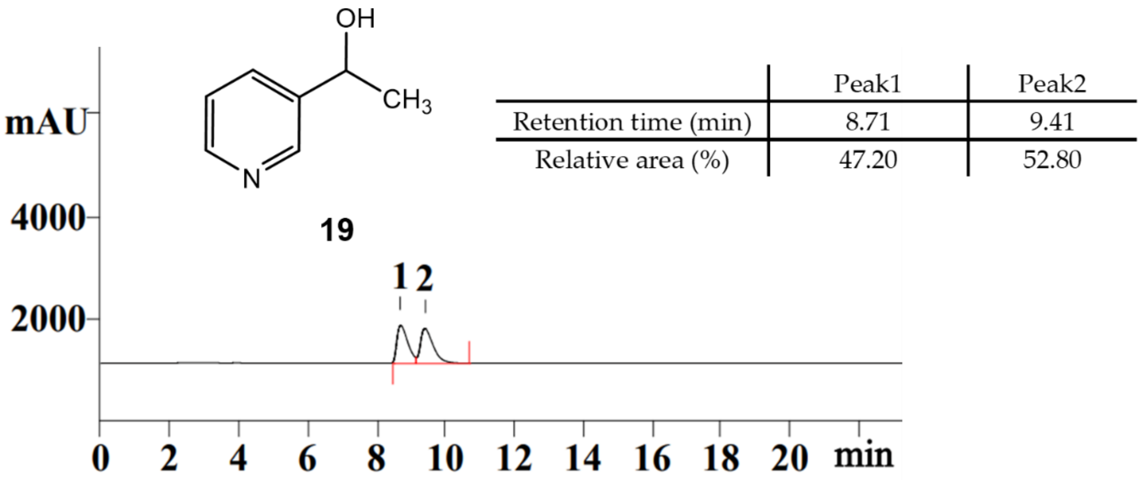

	Peak1	Peak2
Retention time (min)	8.71	9.41
Relative area (%)	47.20	52.80

Figure S8. HPLC chromatogram for the sample (*S*)-**19**, obtained in the autocatalytic reaction (stage 3) with catalyst **19′′**.

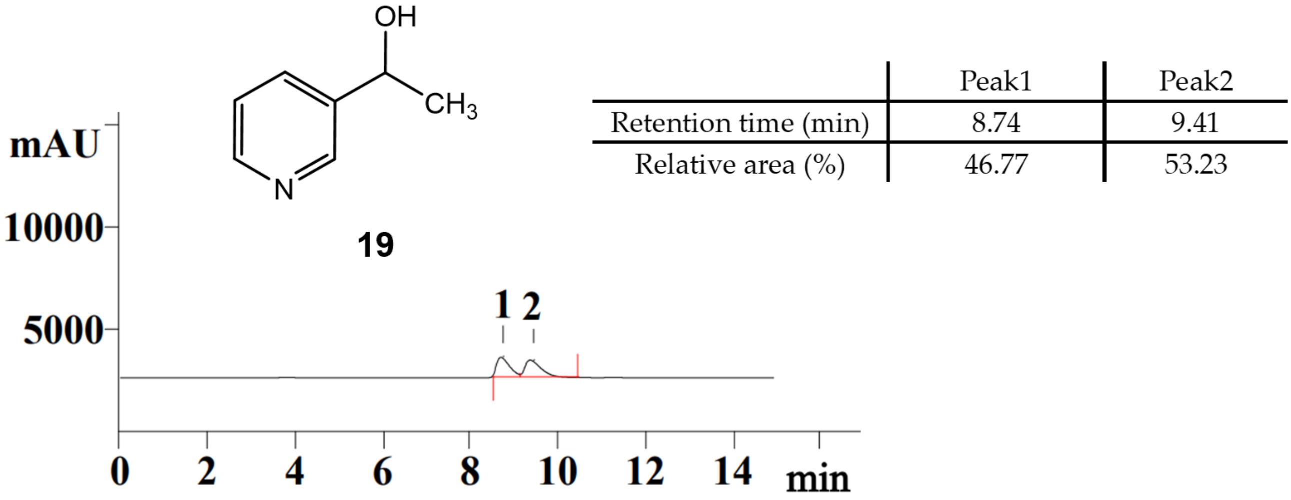

	Peak1	Peak2
Retention time (min)	8.74	9.41
Relative area (%)	46.77	53.23

Figure S9. HPLC chromatogram for the sample (*S*)-**19**, obtained in the autocatalytic reaction with 2% ee catalyst (*S*)-**19**.

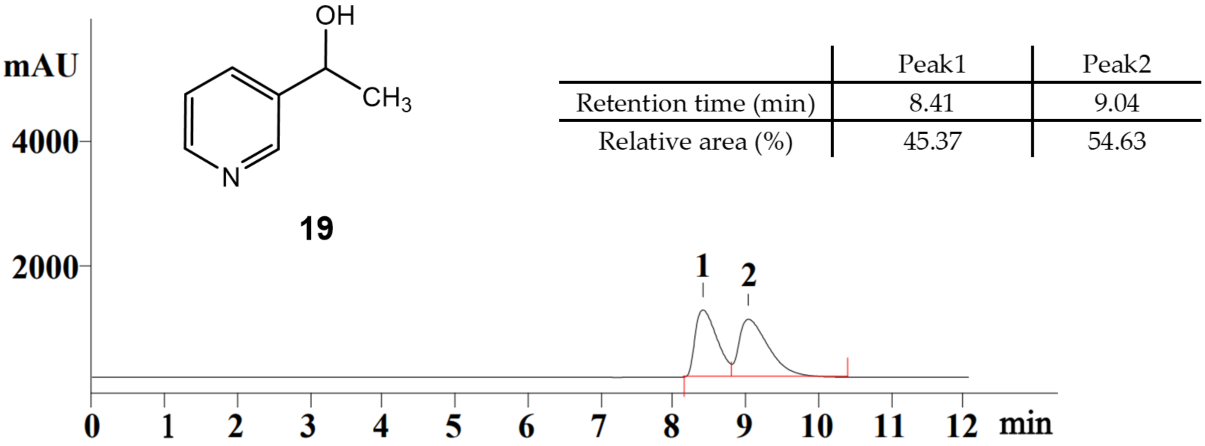

	Peak1	Peak2
Retention time (min)	8.41	9.04
Relative area (%)	45.37	54.63

Figure S10. HPLC chromatogram for the sample (*S*)-**19**, obtained in the autocatalytic reaction with 6% ee catalyst (*S*)-**19**.

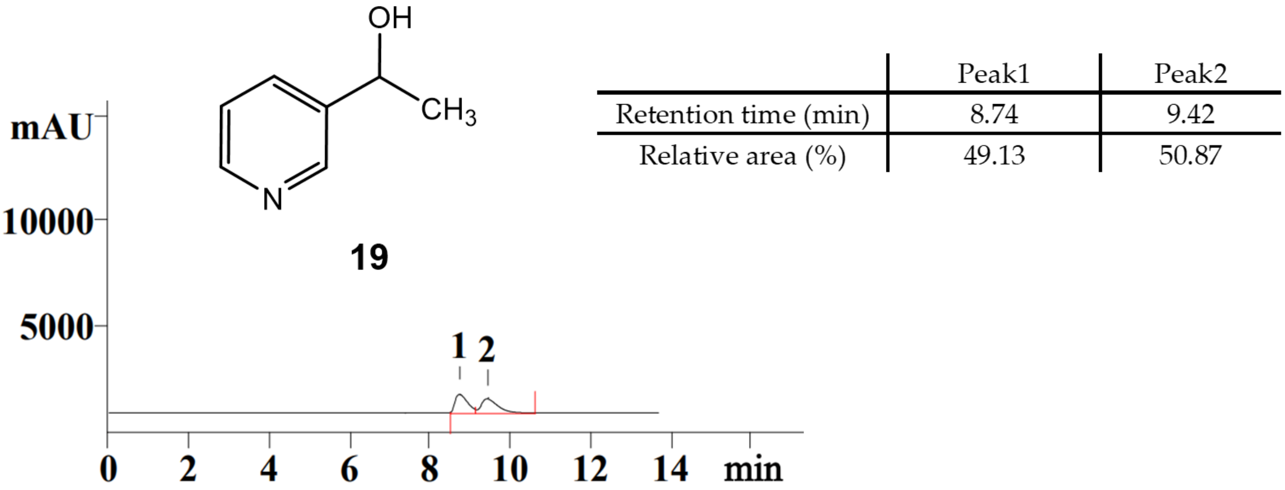

	Peak1	Peak2
Retention time (min)	8.74	9.42
Relative area (%)	49.13	50.87

Figure S11. HPLC chromatogram of the catalyst (*S*)-19 with 2% ee.

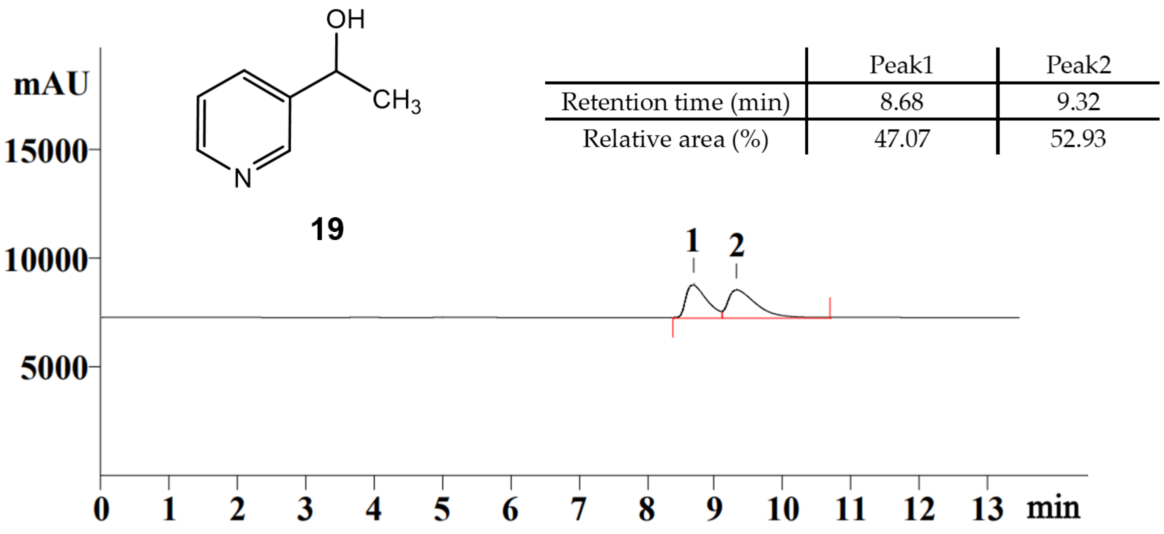

Figure S12. HPLC chromatogram of the catalyst (*S*)-**19** with 6% ee.

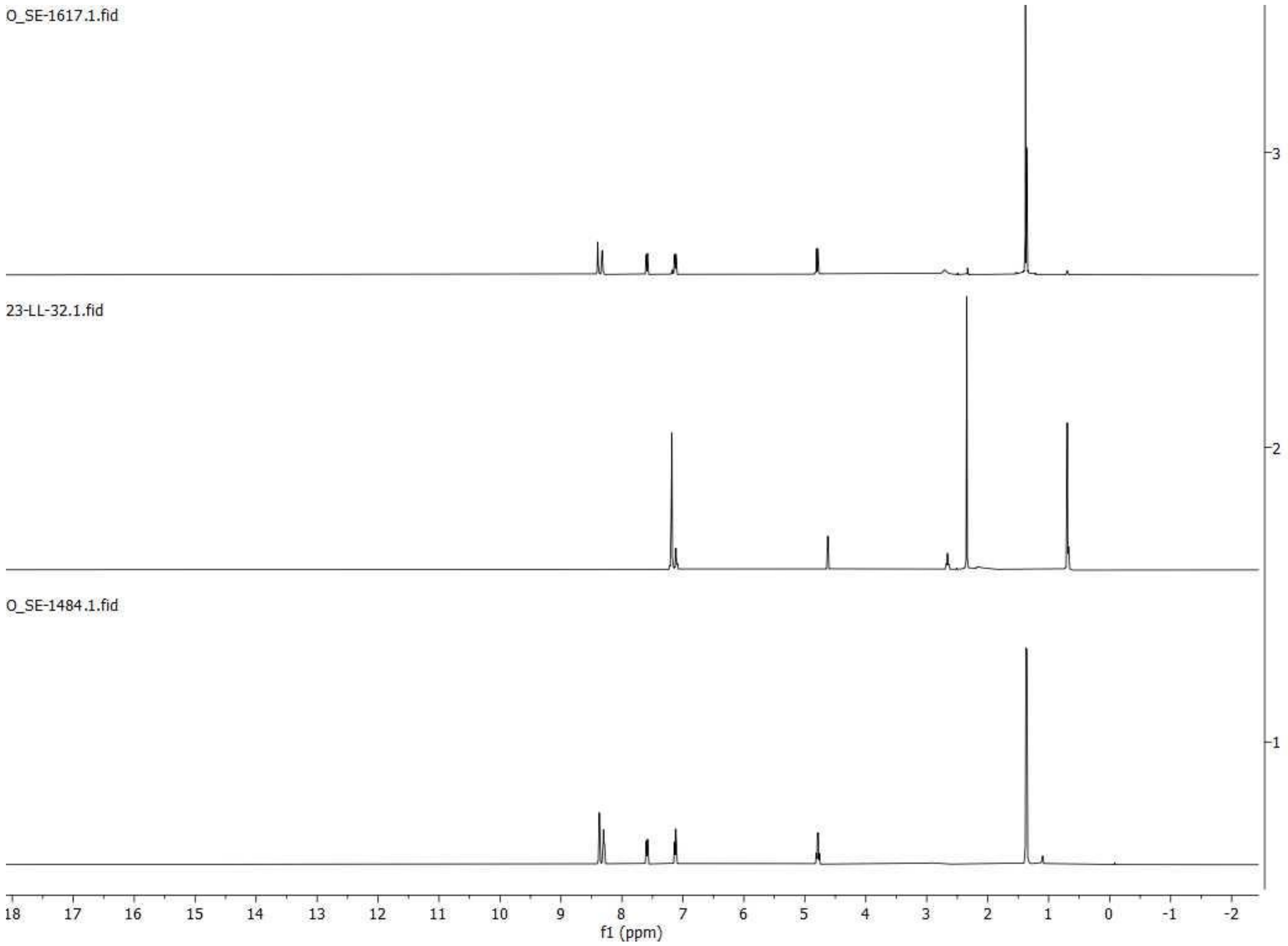

Figure S13: below, ^{1}H NMR spectrum of the alcohol **19** purified for HPLC analysis; middle: ^{1}H NMR spectrum of Ephedrine; top: ^{1}H NMR spectrum of the sample containing the mixture of **19** and ephedrine in a 100:1 ratio. The signals of the ephedrine in mixture are clearly visible.

5. Cartesian Coordinates

3

Sum of electronic and zero-point Energies= -2571.372555
Sum of electronic and thermal Energies= -2571.351382
Sum of electronic and thermal Enthalpies= -2571.350437
Sum of electronic and thermal Free Energies= -2571.425790

Standard orientation:

Center Atomic Atomic Coordinates (Angstroms)
Number Number Type X Y Z
----
1 6 0 3.286244 -1.699982 0.007229
2 1 0 2.718168 -2.038935 0.879845
3 1 0 4.025981 -2.477679 -0.213759
4 6 0 2.349283 -1.452203 -1.199056
5 1 0 2.857319 -1.785290 -2.119854
6 1 0 1.424828 -2.040733 -1.126480
7 6 0 2.123967 0.071034 -1.216582
8 1 0 1.852469 0.391576 -2.235684
9 6 0 3.531367 0.578061 -0.864114
10 1 0 3.575391 1.640471 -0.594390
11 1 0 4.228953 0.431609 -1.707619
12 6 0 3.958242 -0.332322 0.298818
13 1 0 5.046738 -0.410421 0.397411
14 1 0 3.579843 0.077446 1.243640
15 30 0 0.693128 0.687676 0.011297
16 6 0 -0.104919 2.189751 1.031413
17 1 0 0.539615 2.372519 1.904237
18 6 0 -0.251684 3.477225 0.204009
19 1 0 -0.428189 4.352930 0.853132
20 1 0 0.617172 3.714797 -0.422342
21 6 0 -1.555887 1.967106 1.499248
22 1 0 -1.772315 0.935156 1.806943
23 1 0 -1.790756 2.599426 2.370887
24 6 0 -1.514412 3.217993 -0.629654
25 1 0 -1.983598 4.136996 -0.998160
26 1 0 -1.249600 2.619798 -1.512341
27 6 0 -2.443977 2.406383 0.306585
28 1 0 -2.871525 1.543223 -0.216145
29 1 0 -3.289820 3.013366 0.648017
30 7 0 -0.988392 -0.619881 -0.628209
31 6 0 -1.632040 -0.484928 -1.783689
32 6 0 -1.371670 -1.588467 0.215563
33 6 0 -2.698690 -1.301806 -2.149955
34 1 0 -1.281742 0.309097 -2.436953
35 6 0 -2.430703 -2.445795 -0.064259
36 6 0 -3.106534 -2.296215 -1.271686
37 1 0 -3.193345 -1.153299 -3.103246
38 1 0 -2.722632 -3.217413 0.638978
39 1 0 -3.935843 -2.950068 -1.520731
40 6 0 -0.552509 -1.671508 1.471453
41 8 0 0.407232 -0.927558 1.601606

42 6 0 -0.923528 -2.665359 2.532200
43 1 0 -0.236867 -2.567476 3.373765
44 1 0 -1.950131 -2.495889 2.875298
45 1 0 -0.868356 -3.685664 2.136124

TS1

Sum of electronic and zero-point Energies= -2571.348478
Sum of electronic and thermal Energies= -2571.328753
Sum of electronic and thermal Enthalpies= -2571.327809
Sum of electronic and thermal Free Energies= -2571.397304
$\nu = i666.37$
Standard orientation:

Center Atomic Atomic Coordinates (Angstroms)
Number Number Type X Y Z
----
1 6 0 -3.351799 1.434331 -0.503151
2 1 0 -2.441149 2.044206 -0.486296
3 1 0 -4.150769 2.067714 -0.903905
4 6 0 -3.139768 0.167393 -1.357909
5 1 0 -4.100105 -0.114202 -1.822131
6 1 0 -2.436188 0.335320 -2.184413
7 6 0 -2.693251 -0.904566 -0.348039
8 1 0 -2.872631 -1.909142 -0.757456
9 6 0 -3.660376 -0.620163 0.814387
10 1 0 -3.383276 -1.098692 1.761171
11 1 0 -4.677378 -0.968915 0.565515
12 6 0 -3.669891 0.916718 0.925855
13 1 0 -4.625271 1.297489 1.303031
14 1 0 -2.895574 1.247033 1.627808
15 30 0 -0.792833 -0.751225 0.176568
16 6 0 0.720556 -2.171275 -0.328274
17 1 0 0.169236 -3.050977 0.007068
18 6 0 0.970006 -2.051761 -1.839829
19 1 0 1.124737 -3.051949 -2.272206
20 1 0 0.153108 -1.592552 -2.410129
21 6 0 1.964461 -1.916456 0.343576
22 1 0 1.871386 -0.814397 1.013845
23 1 0 2.225962 -2.530214 1.210624
24 6 0 2.270861 -1.237492 -1.940533
25 1 0 2.823759 -1.433019 -2.864330
26 1 0 2.051408 -0.165086 -1.911700
27 6 0 3.055966 -1.640339 -0.684059
28 1 0 3.787979 -0.889430 -0.366587
29 1 0 3.602577 -2.576371 -0.861818
30 7 0 0.331338 1.045151 -0.255680
31 6 0 0.322260 1.849113 -1.325573
32 6 0 1.316184 1.168570 0.650985
33 6 0 1.289325 2.817135 -1.540112
34 1 0 -0.494548 1.700028 -2.025570
35 6 0 2.339096 2.105934 0.497932
36 6 0 2.317043 2.944509 -0.606172
37 1 0 1.238544 3.455161 -2.415187
38 1 0 3.129470 2.182108 1.236214
39 1 0 3.092116 3.692750 -0.740419

40 6 0 1.233252 0.137208 1.742986
41 8 0 0.059061 -0.361415 1.928829
42 6 0 2.159177 0.247413 2.934998
43 1 0 2.058215 -0.657146 3.539748
44 1 0 3.207696 0.358368 2.642120
45 1 0 1.875223 1.106319 3.554693

8

Sum of electronic and zero-point Energies= -2571.395752
Sum of electronic and thermal Energies= -2571.373913
Sum of electronic and thermal Enthalpies= -2571.372969
Sum of electronic and thermal Free Energies= -2571.451735
Standard orientation:

Center Atomic Atomic Coordinates (Angstroms)
Number Number Type X Y Z
----
1 6 0 -4.065932 0.113171 -1.240603
2 1 0 -4.408536 1.149020 -1.146939
3 1 0 -4.458834 -0.264545 -2.190437
4 6 0 -2.524335 0.005685 -1.227762
5 1 0 -2.094171 0.098981 -2.231611
6 1 0 -2.109739 0.828663 -0.629732
7 6 0 -2.218502 -1.341351 -0.552623
8 1 0 -2.497667 -2.130137 -1.270687
9 6 0 -3.270786 -1.360491 0.568872
10 1 0 -2.946750 -0.715317 1.396432
11 1 0 -3.437123 -2.358303 0.990381
12 6 0 -4.552951 -0.775479 -0.066562
13 1 0 -5.188137 -1.584602 -0.442272
14 1 0 -5.152778 -0.213436 0.656973
15 30 0 -0.368473 -1.602036 0.007436
16 6 0 1.500676 -2.136570 0.174297
17 1 0 1.526720 -3.181794 0.527786
18 6 0 2.316693 -2.099813 -1.131051
19 1 0 2.006104 -2.857144 -1.859536
20 1 0 2.205701 -1.123156 -1.624662
21 6 0 2.405985 -1.329719 1.116259
22 1 0 2.299225 -0.255547 0.913162
23 1 0 2.166880 -1.474403 2.175235
24 6 0 3.787843 -2.275941 -0.693375
25 1 0 4.066792 -3.333898 -0.741248
26 1 0 4.481109 -1.736109 -1.346554
27 6 0 3.846588 -1.770768 0.772066
28 1 0 4.568925 -0.957903 0.900259
29 1 0 4.160616 -2.580698 1.439031
30 6 0 1.380839 2.217731 -1.786991
31 6 0 0.425031 2.191360 0.409687
32 1 0 1.680377 1.706124 -2.697720
33 6 0 0.657648 3.564645 0.465775
34 6 0 1.248058 4.182427 -0.632841
35 1 0 0.391910 4.156382 1.334420
36 1 0 1.441038 5.251968 -0.617355
37 6 0 -0.200563 1.426768 1.536575
38 8 0 -0.384128 0.223377 1.430095

39 6 0 -0.595814 2.154273 2.788255
40 1 0 -1.333814 2.929540 2.558057
41 1 0 -1.020898 1.445781 3.498488
42 1 0 0.273166 2.647209 3.235613
43 6 0 0.797082 1.508901 -0.746719
44 1 0 0.629957 0.442365 -0.817879
45 7 0 1.607396 3.533721 -1.741801

TS2

Sum of electronic and zero-point Energies= -2571.368362
Sum of electronic and thermal Energies= -2571.348353
Sum of electronic and thermal Enthalpies= -2571.347409
Sum of electronic and thermal Free Energies= -2571.418965
$\nu = i$ 687.37
Standard orientation:

Center Atomic Atomic Coordinates (Angstroms)
Number Number Type X Y Z
----
1 6 0 1.360258 3.235057 -0.937674
2 1 0 2.321230 3.346692 -0.428019
3 1 0 1.439822 3.748370 -1.905248
4 6 0 0.958120 1.791505 -1.208636
5 1 0 1.497842 1.228916 -1.972642
6 1 0 1.531945 1.166406 -0.184154
7 6 0 -0.461368 1.663447 -1.097068
8 1 0 -0.947289 1.097314 -1.895278
9 6 0 -1.032190 3.054199 -0.775049
10 1 0 -1.911923 3.057988 -0.122259
11 1 0 -1.329118 3.561023 -1.704270
12 6 0 0.164451 3.779871 -0.146033
13 1 0 0.081466 4.868410 -0.192488
14 1 0 0.258748 3.491603 0.907513
15 30 0 -1.100557 0.444173 0.471983
16 6 0 -2.814845 -0.435757 0.826665
17 1 0 -3.300526 0.036528 1.690364
18 6 0 -3.737246 -0.398248 -0.403917
19 1 0 -4.768820 -0.658631 -0.119513
20 1 0 -3.784144 0.575151 -0.906105
21 6 0 -2.680880 -1.953584 1.043780
22 1 0 -1.868091 -2.247617 1.718115
23 1 0 -3.608936 -2.358353 1.476659
24 6 0 -3.175340 -1.498043 -1.325633
25 1 0 -3.952490 -1.952514 -1.947211
26 1 0 -2.434038 -1.073262 -2.013139
27 6 0 -2.499909 -2.525893 -0.377145
28 1 0 -1.435240 -2.621529 -0.620501
29 1 0 -2.933153 -3.525884 -0.472950
30 6 0 1.273017 -1.914724 0.380966
31 6 0 3.632538 -2.196220 -0.922155
32 6 0 2.124781 -0.812503 0.332720
33 6 0 3.336774 -0.968537 -0.343775
34 1 0 4.572485 -2.334851 -1.451460
35 1 0 4.044353 -0.150987 -0.432941
36 1 0 0.327881 -1.855817 0.909661

37 6 0 1.688360 0.503989 0.939509
38 8 0 0.514129 0.512046 1.501125
39 6 0 2.753591 1.331862 1.637865
40 1 0 3.055728 0.827114 2.561407
41 1 0 3.643902 1.485371 1.023757
42 1 0 2.333250 2.304934 1.901821
43 6 0 1.663161 -3.101181 -0.231744
44 1 0 1.009225 -3.969815 -0.197336
45 7 0 2.819987 -3.257080 -0.878335

10
Sum of electronic and zero-point Energies= -2571.394233
Sum of electronic and thermal Energies= -2571.373560
Sum of electronic and thermal Enthalpies= -2571.372616
Sum of electronic and thermal Free Energies= -2571.446527
Standard orientation:

Center Atomic Atomic Coordinates (Angstroms)
Number Number Type X Y Z
----
1 6 0 1.477368 -3.242335 -0.189339
2 1 0 0.894383 -3.139405 0.731246
3 1 0 1.432330 -4.296411 -0.480840
4 6 0 0.914693 -2.323884 -1.297797
5 1 0 1.003187 -2.836171 -2.268209
6 1 0 -0.151550 -2.113878 -1.156637
7 6 0 1.814637 -1.076681 -1.272675
8 1 0 1.784090 -0.573419 -2.249992
9 6 0 3.196367 -1.719971 -1.051919
10 1 0 3.991160 -1.017758 -0.773946
11 1 0 3.533395 -2.232011 -1.967407
12 6 0 2.936579 -2.768358 0.042168
13 1 0 3.656790 -3.591923 0.014105
14 1 0 3.027487 -2.302181 1.031149
15 30 0 1.411952 0.272625 0.093836
16 6 0 1.422005 1.947403 1.105346
17 1 0 1.847343 1.750850 2.099752
18 6 0 2.201694 3.067719 0.396146
19 1 0 2.407048 3.898847 1.090339
20 1 0 3.166678 2.758256 -0.022101
21 6 0 0.031457 2.597194 1.250812
22 1 0 -0.777533 1.879967 1.431056
23 1 0 0.022564 3.294517 2.102181
24 6 0 1.226689 3.550847 -0.688058
25 1 0 1.432264 4.573485 -1.019214
26 1 0 1.313896 2.908378 -1.573624
27 6 0 -0.181387 3.398645 -0.057344
28 1 0 -0.860360 2.879418 -0.742390
29 1 0 -0.636087 4.372776 0.147953
30 7 0 -2.619682 0.728834 -1.687473
31 6 0 -3.932353 0.479814 -1.635981
32 6 0 -1.864084 0.190218 -0.736546
33 6 0 -4.530050 -0.309466 -0.657911
34 1 0 -4.535516 0.933693 -2.418126

35 6 0 -2.355566 -0.608071 0.300547
36 6 0 -3.727583 -0.865665 0.328117
37 1 0 -5.600356 -0.479739 -0.671587
38 1 0 -4.167736 -1.481714 1.105402
39 1 0 -0.799603 0.391146 -0.788769
40 6 0 -1.405326 -1.135999 1.318135
41 8 0 -0.231115 -0.788191 1.310250
42 6 0 -1.902415 -2.102320 2.355516
43 1 0 -2.681724 -1.638098 2.968700
44 1 0 -2.337912 -2.985106 1.876716
45 1 0 -1.072455 -2.403093 2.994189

TS3

Sum of electronic and zero-point Energies= -2571.366809
Sum of electronic and thermal Energies= -2571.346535
Sum of electronic and thermal Enthalpies= -2571.345591
Sum of electronic and thermal Free Energies= -2571.419736
$\nu = i\,773.18$
Standard orientation:

Center Atomic Atomic Coordinates (Angstroms)
Number Number Type X Y Z
----
1 6 0 -2.277001 -2.788759 -0.796560
2 1 0 -3.186468 -2.636779 -0.207293
3 1 0 -2.565945 -3.300381 -1.724085
4 6 0 -1.556282 -1.505119 -1.184572
5 1 0 -2.007118 -0.830975 -1.914585
6 1 0 -1.894815 -0.709483 -0.130681
7 6 0 -0.155066 -1.721847 -1.174053
8 1 0 0.428495 -1.274345 -1.979271
9 6 0 0.108348 -3.190871 -0.805443
10 1 0 1.007237 -3.370884 -0.204453
11 1 0 0.222860 -3.790990 -1.719534
12 6 0 -1.183948 -3.593610 -0.076006
13 1 0 -1.370159 -4.670346 -0.092438
14 1 0 -1.126788 -3.277720 0.970458
15 30 0 0.837700 -0.759466 0.400194
16 6 0 2.762705 -0.528500 0.623053
17 1 0 3.156686 -1.334562 1.256911
18 6 0 3.461939 -0.509116 -0.742092
19 1 0 4.551061 -0.630249 -0.626977
20 1 0 3.124803 -1.293627 -1.430596
21 6 0 3.158709 0.851776 1.198538
22 1 0 2.483615 1.222880 1.978520
23 1 0 4.157330 0.798340 1.656865
24 6 0 3.159370 0.899894 -1.267968
25 1 0 3.848667 1.218832 -2.055597
26 1 0 2.151273 0.920853 -1.703252
27 6 0 3.217730 1.810919 -0.017258
28 1 0 2.393325 2.531004 -0.008752
29 1 0 4.140617 2.399344 0.001378
30 7 0 -0.440619 3.391858 -0.004127
31 6 0 -1.386922 3.822654 -0.839344
32 6 0 -0.612226 2.192151 0.561922

33 6 0 -2.528738 3.084757 -1.147828
34 1 0 -1.217144 4.790798 -1.305191
35 6 0 -1.716498 1.372627 0.326826
36 6 0 -2.693244 1.842701 -0.555002
37 1 0 -3.259883 3.477937 -1.845447
38 1 0 -3.558138 1.227876 -0.794935
39 1 0 0.153524 1.863745 1.264904
40 6 0 -1.776704 0.002678 0.958895
41 8 0 -0.661589 -0.404567 1.506004
42 6 0 -3.039485 -0.339007 1.731181
43 1 0 -3.068487 0.251280 2.653362
44 1 0 -3.950233 -0.128333 1.166562
45 1 0 -3.024390 -1.396684 2.003424
